# PPARγ Signaling in the Central Nervous System: A Review of the Implications in Synaptic Plasticity, Neuroinflammatory and Neoplastic Processes

**DOI:** 10.1007/s11064-026-04908-2

**Published:** 2026-10-12

**Authors:** Vanessa Camylla Bernardo de Oliveira Maciel, Josenilda de Moura Arruda, Ryan Cordeiro Silva, Michelle Melgarejo da Rosa

**Affiliations:** 1 Laboratory of Immunomodulation and New Therapeutic Approaches (LINAT), Nucleus of Research in Therapeutic Innovation Suely Galdino (NUPIT-SG), Biosciences Center, Federal University of Pernambuco, Recife, Brazil; 2 Departament of Biochemistry, UFPE, A. Prof. Moraes Rego, 1235, Cidade Universitária, Recife, PE 50670-901 Brazil

**Keywords:** PPARγ, Neuroinflammation, Synaptic plasticity, Synaptic remodeling, Thiazolidinediones

## Abstract

Peroxisome proliferator activated receptor gamma (PPARγ) is a ligand activated nuclear receptor traditionally associated with lipid and glucose metabolism. Emerging evidence, however, identifies PPARγ as a central immunometabolic regulator in the central nervous system (CNS), coordinating mitochondrial function, neuroinflammation, synaptic plasticity, and neurovascular integrity. Beyond its classical metabolic actions, PPARγ regulates microglial activation, astrocytic inflammatory responses, oxidative stress, and mitochondrial biogenesis, thereby influencing neuronal resilience and circuit stability. This narrative review synthesizes knowledge of PPARγ structure, activation, CNS distribution, endogenous ligands, and synthetic agonists, including thiazolidinediones and selective modulators. We highlight how PPARγ controls transcriptional programs related to energy homeostasis, inflammatory signaling, including NF κB dependent pathways, and blood brain barrier function. Attention is devoted to synaptic remodeling and plasticity, positioning PPARγ as a molecular bridge between metabolic state and neuronal adaptability. We propose that PPARγ signaling dysregulation represents a convergent mechanism in neurodegenerative disorders, including Alzheimer’s disease, Parkinson’s disease, and adrenoleukodystrophy; psychiatric conditions, such as depression and anxiety; and neurooncological processes. Integrating evidence across these conditions supports PPARγ as a master regulator of neuroimmune metabolic coupling in the brain. Finally, we discuss translational challenges and opportunities, including brain penetrant, functionally selective ligands, clarification of cell type specific signaling, and expansion of rigorous clinical trials. Integrating mechanistic, preclinical, and clinical trial evidence, this review critically examines the therapeutic potential of PPARγ across neurodegenerative, psychiatric, and neurooncological CNS conditions, while identifying priorities for future therapeutic development in diverse brain disorders.

## Introduction

Brain disorders, including neurodegenerative diseases, psychiatric conditions, and neuro-oncological processes, are increasingly recognized as disorders of disrupted neuroimmune–metabolic homeostasis rather than purely neuronal pathologies [1]. Converging evidence implicates mitochondrial dysfunction, chronic neuroinflammation, oxidative stress [2–5], and impaired neurovascular integrity [6] as shared mechanisms underlying cognitive decline, mood dysregulation, and neuronal vulnerability [7–10]. Identifying molecular regulators capable of integrating metabolic and inflammatory signaling within the central nervous system (CNS) has therefore become a major priority in translational neuroscience.

Peroxisome proliferator-activated receptors (PPARs) are ligand-activated transcription factors belonging to the class III nuclear receptor superfamily that function as metabolic sensors and transcriptional regulators [11]. Upon ligand binding, PPARs heterodimerize with retinoid X receptors (RXRα, RXRβ, RXRγ) and recruit transcriptional coactivators such as PPARγ coactivator-1 (PGC-1) [12, 13] steroid receptor coactivators (SRC) [14], cAMP response element-binding protein (CREB) and its transcriptional coactivators CREB-binding protein (CBP) and p300 [15], initiating gene programs involved in lipid metabolism, glucose homeostasis, cellular differentiation, and inflammatory modulation [12, 16]. In addition to classical transactivation, PPARs exert anti-inflammatory actions through transrepression mechanisms by interacting with nuclear co-repressors (NCoR) and silencing mediator of retinoid and thyroid receptors (SMRT), thereby inhibiting pro-inflammatory transcriptional cascades [12, 17].

Three isoforms, PPARα, PPARβ/δ, and PPARγ, are encoded by distinct genes and display overlapping yet functionally specialized roles in metabolic regulation, cell proliferation, and differentiation [18–20]. Dysregulated PPAR signaling has been strongly associated with metabolic disorders, including type 2 diabetes, dyslipidemia, and obesity [21–23]. However, beyond their well-established peripheral roles, PPARs are increasingly recognized as critical regulators within the CNS [24].

Although PPARβ/δ and PPARα exhibit widespread cerebral expression [25, 26], PPARγ has drawn particular attention due to its potent immunomodulatory and mitochondrial regulatory properties [27, 28]. PPARγ is expressed in neurons, astrocytes, microglia, and endothelial cells across regions implicated in cognition and emotional regulation, including the cortex, hippocampus, and striatum [21–31].

Within the CNS, PPARγ regulates transcriptional programs linked to mitochondrial biogenesis, oxidative stress buffering, excitatory neurotransmission, and inflammatory signaling [32–34]. Through these actions, PPARγ influences synaptic remodeling [35], neuronal survival [36], and glial activation states [37], positioning it at the interface between metabolic status and neural plasticity. PPARγ operates as one node within a broader metabolic network governing microglial polarization, alongside key regulators of cellular energy metabolism, growth, and adaptation to hypoxic conditions, including AMP-activated protein kinase (AMPK), mechanistic target of rapamycin (mTOR), and hypoxia-inducible factor 1-alpha (HIF-1α) [38].

Neuroinflammation and metabolic stress are now considered central drivers of neurodegenerative and psychiatric disorders [5, 34, 39, 40]. Microglial activation, astrocytic reactivity, blood–brain barrier (BBB) dysfunction, and impaired mitochondrial bioenergetics converge to disrupt synaptic integrity and circuit stability [41–43]. Notably, PPARγ signaling intersects with key inflammatory pathways, including nuclear factor kappa B (NF-κB)-dependent transcription, while also modulating antioxidant defenses and cellular energy metabolism [44]. These properties suggest that PPARγ functions not merely as a metabolic regulator, but as a molecular integrator of neuroimmune and bioenergetic signaling in the brain.

Pharmacological activation of PPARγ, particularly through thiazolidinediones (TZDs) and emerging selective PPARγ modulators (SPPARMs), has shown neuroprotective and anti-inflammatory effects in preclinical models [23, 35, 44, 45]. However, clinical translation remains inconsistent, highlighting the need for a deeper mechanistic understanding of cell type–specific signaling, ligand selectivity, brain penetrance, and context-dependent responses.

Several reviews have already discussed PPARγ in the context of neurodegeneration, neuroinflammation, and metabolic regulation. However, most focus on a single disease area or consider PPARγ primarily as a peripheral metabolic regulator, with comparatively limited attention to its functions within the CNS [11, 24]. Consequently, the mechanistic links between mitochondrial function, synaptic plasticity, and neuroimmune signaling have rarely been examined within a unified framework, nor has their disruption been systematically compared across neurodegenerative, psychiatric, and neuro-oncological conditions.

Moreover, existing reviews have largely summarized preclinical evidence without critically addressing why PPARγ-targeted clinical trials have yielded limited success. This review addresses these gaps by positioning synaptic plasticity as a mechanistic interface between the metabolic and immunomodulatory actions of PPARγ and neuronal function. We further integrate evidence across neurodegenerative, psychiatric, and neuro-oncological conditions and compare preclinical rationale with clinical outcomes to examine the limitations of first-generation agonists and their implications for the development of next-generation CNS-SPPARMs.

## PPARγ Structure, Isoform Diversity, and Ligand-Dependent Modulation

### Structural Organization and Isoform-Specific Features of PPARγ

PPARs share a conserved modular architecture typical of nuclear receptor superfamily members, comprising six functional domains (A–F) as defined by structural and crystallographic analyses [46]. This modular organization underlies their ability to integrate ligand sensing with transcriptional control [47].

The N-terminal A/B domain contains the ligand-independent activation function 1 (AF-1), which contributes to basal transcriptional activity and allows context-dependent modulation through post-translational modifications. The central C domain encodes the highly conserved DNA-binding domain (DBD), characterized by two zinc finger motifs that recognize peroxisome proliferator response elements (PPREs) within target gene promoters [48, 49].

**Fig. 1:**
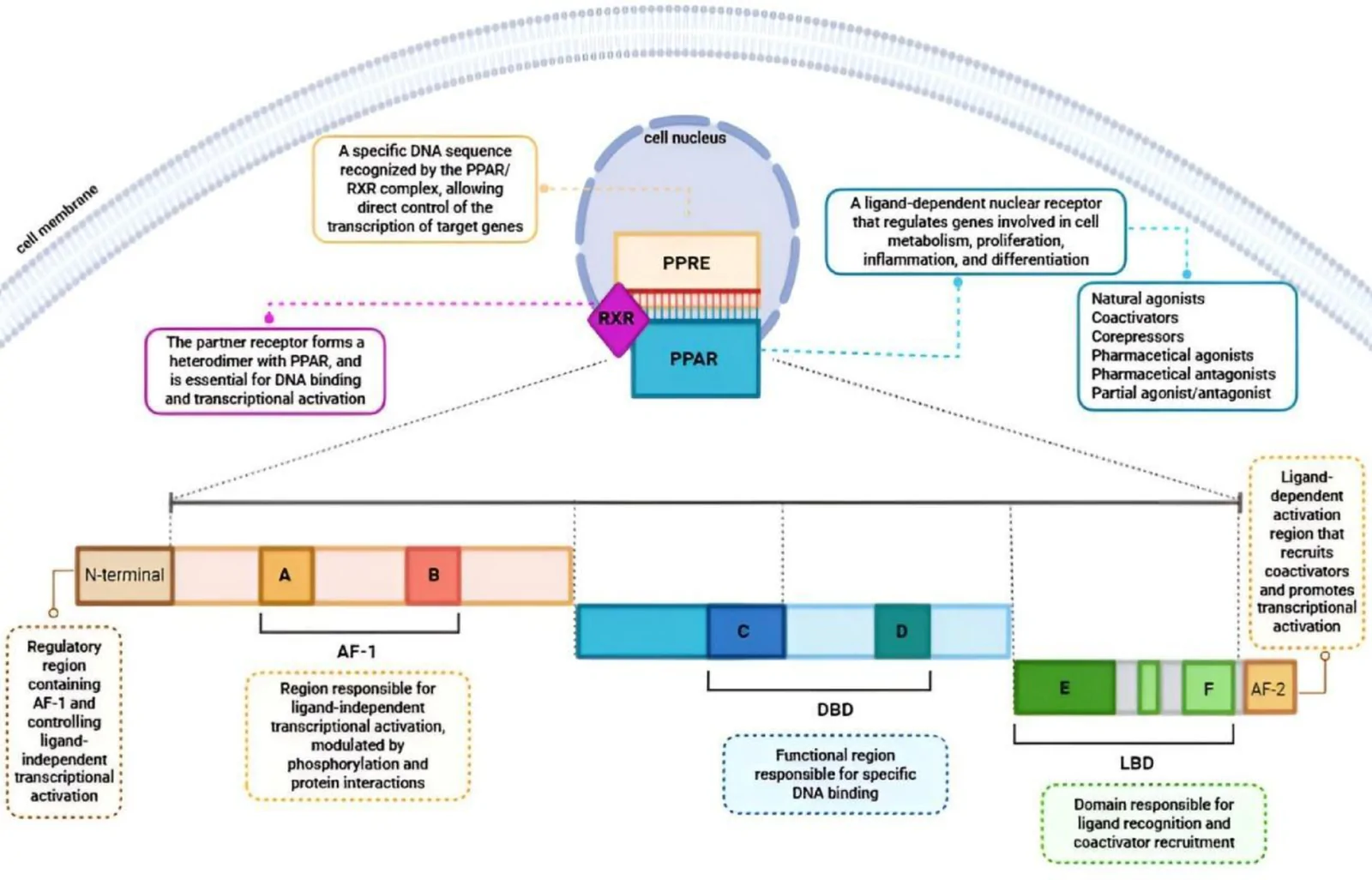
Structural organization and transcriptional mechanism of the Peroxisome Proliferator-Activated Receptor (PPAR). The diagram illustrates the functional dynamics of PPAR as a ligand-dependent nuclear receptor. In the cell nucleus, PPAR forms a heterodimer with the Retinoid X Receptor (RXR) to bind specifically to the Peroxisome Proliferator Response Element (PPRE), a conserved DNA sequence that allows direct transcriptional control of target genes involved in metabolism and inflammation. The molecular architecture of the receptor is composed of distinct functional domains: the N-terminal region (A/B), which contains the AF-1 for ligand-independent activation; the DBD - C/D, responsible for high-affinity recognition of the PPRE; and the LBD - E/F. The LBD facilitates the binding of natural and pharmaceutical agonists, dimer formation, and houses the AF-2, which is essential for the ligand-dependent recruitment of coactivators to promote gene transcription

Adjacent to the DBD, the D (hinge) domain confers conformational flexibility, enabling interaction with cofactors and facilitating nuclear localization. The E/F region constitutes the ligand-binding domain (LBD), a multifunctional domain responsible for ligand specificity, heterodimerization with retinoid X receptors (RXRs), and recruitment of transcriptional coregulators. Within the LBD, the activation function 2 (AF-2) helix undergoes ligand-induced conformational rearrangement, stabilizing coactivator binding through short leucine-rich amino acid motifs and enabling assembly of transcriptional complexes involving CBP/p300 and SRC family members [48, 50]. In the absence of activating ligands, PPARs can associate with corepressors such as NCoR and SMRT, thereby maintaining selected target genes in a transcriptionally repressed state (Fig. 1).

This AF-2–dependent selectivity in coactivator recruitment is the structural basis for a defining feature of PPARγ biology, where the same receptor can drive opposing transcriptional programs in metabolic, anti-inflammatory, or pro-differentiation pathways, depending on which ligand engages it and which cell type expresses it. Structural flexibility, in this sense, is the mechanistic root of PPARγ’s functional versatility, rather than a source of inconsistency to be explained away.

Although structurally homologous, PPARα, PPARβ/δ, and PPARγ exhibit distinct tissue distributions, ligand specificities, and transcriptional outputs [51]. PPARα, expressed predominantly in metabolically active peripheral tissues such as liver and skeletal muscle, regulates fatty acid β-oxidation and is the pharmacological target of fibrates in hypertriglyceridemia management [52, 53]. PPARβ/δ, by contrast, is more ubiquitously expressed, including in CNS neurons and glia, and has been implicated in neuroinflammatory modulation, though its role in the CNS remains considerably less characterized than that of PPARγ [29, 30, 39, 54].

In contrast, PPARγ displays distinctive structural and functional properties that make it particularly relevant for CNS immunometabolic regulation [55, 56]. Three isoforms (PPARγ1, PPARγ2, and PPARγ3) arise from alternative promoter usage and splicing [57]. Notably, PPARγ possesses a comparatively large and flexible ligand-binding pocket (LBP), enabling recognition of structurally diverse endogenous ligands, including oxidized lipids and fatty acid derivatives, as well as synthetic TZDs such as troglitazone (TGZ), rosiglitazone (RGZ), ciglitazone (CGZ), and pioglitazone (PIO) [45]. This structural adaptability underlies its pharmacological versatility but also contributes to differential transcriptional responses depending on ligand conformation and cofactor recruitment [58].

### Endogenous Ligands: Lipid-Derived Signals Linking Metabolism and Inflammation

Among endogenous activators, polyunsaturated fatty acids and their oxidized derivatives are the most extensively studied [59, 60]. Fatty acids such as arachidonic acid, linoleic acid, α-linolenic acid, and eicosapentaenoic acid can bind PPARγ at micromolar concentrations, suggesting that physiological fluctuations in lipid metabolism may directly influence receptor activation [59–61].

Linoleic acid (C18:2n-6), an essential dietary fatty acid, and its derivatives, including hydroxyoctadecadienoic acids (9-HODE and 13-HODE), have been shown to transactivate PPARγ in a dose-dependent manner [62]. HODE metabolites regulate inflammatory signaling and apoptosis in immune cells, with pro-apoptotic effects in THP-1 monocytes mediated via PPARγ activation [62, 63]. Arachidonic acid–derived eicosanoids represent another critical class of endogenous modulators. Among them, 15-deoxy-Δ12,14-prostaglandin J2 (15d-PGJ2), generated through sequential conversion of PGD2, is considered a high-affinity endogenous PPARγ ligand [64, 65]. 15d-PGJ2 exerts potent anti-inflammatory effects, including suppression of pro-inflammatory cytokine production and attenuation of ischemia–reperfusion injury [64, 66, 67]. In addition to classical PPARγ-dependent transcriptional regulation, 15d-PGJ2 can induce reactive oxygen species production, activate p38 MAPK, and trigger apoptosis through partially PPARγ-independent mechanisms [64, 66, 68, 69]. These findings highlight how oxidized lipid species generated during inflammatory or oxidative stress conditions can act as endogenous PPARγ ligands, linking lipid peroxidation to transcriptional regulation.

Leukotrienes, including LTE4, have also been reported to interact with PPARγ. LTE4, produced by mast cells and other immune cells, can modulate inflammatory signaling and cyclooxygenase activity [70]. Notably, Paruchuri et al. [71] showed that both 15d-PGJ2 and LTE4 induced extracellular signal-regulated kinase (ERK) phosphorylation, whereas the synthetic agonist rosiglitazone (RGZ) failed to reproduce this response. These findings suggest that endogenous and synthetic ligands may stabilize distinct PPARγ conformations and generate different coregulator-recruitment patterns, thereby producing divergent downstream signaling. Such differences are consistent with ligand-biased PPARγ signaling, in which synthetic agonists may not fully reproduce the transcriptional and non-transcriptional responses elicited by endogenous ligands. Consequently, ligand-dependent differences in receptor conformation and signaling may contribute to context-dependent pharmacological responses to TZDs, although their specific impact on clinical outcomes remains to be established.

**Fig. 2:**
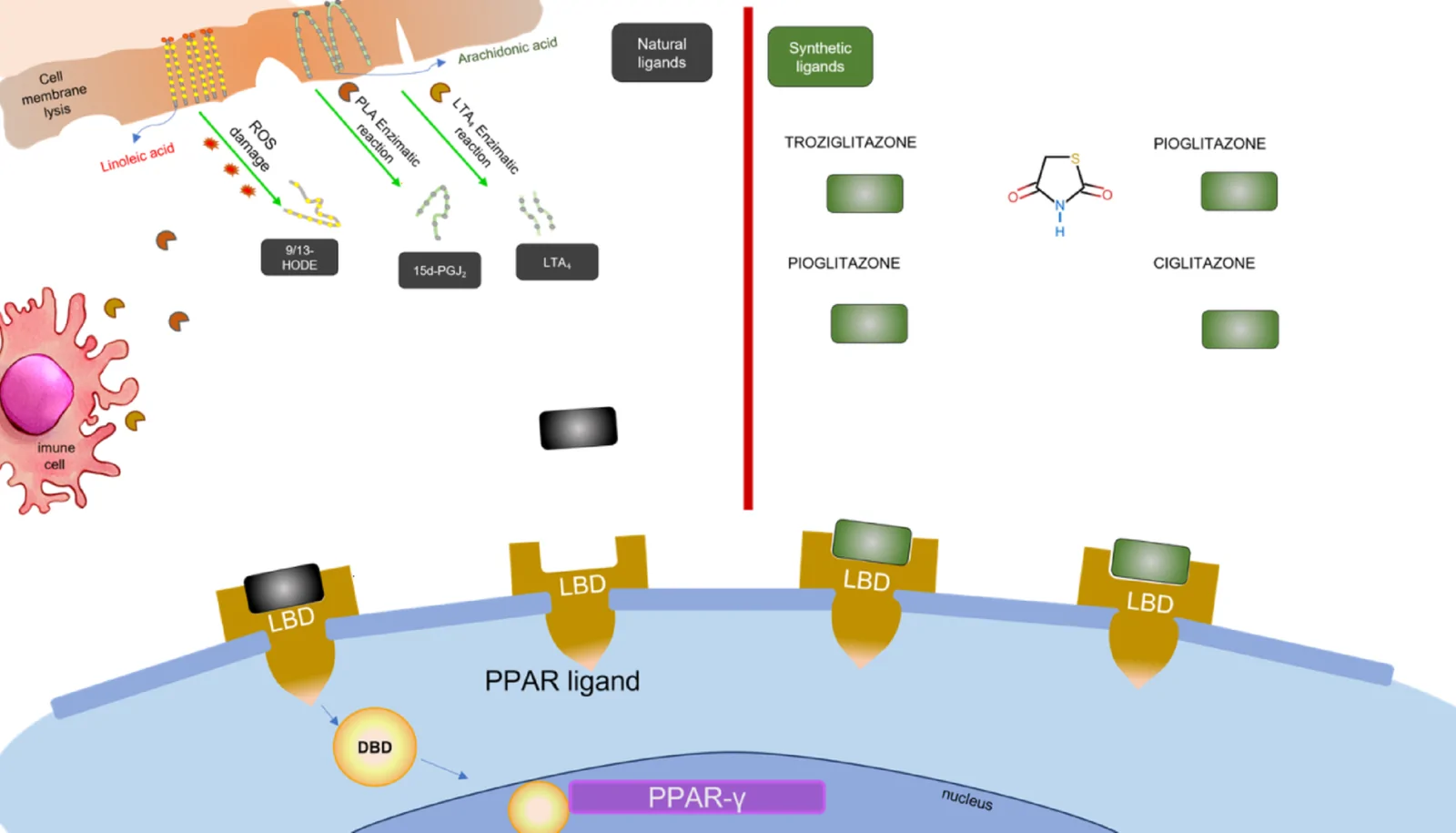
Activation of PPAR-γ by natural and synthetic ligands. Lipid-derived metabolites act as endogenous ligands, while TZDs represent synthetic agonists. Ligand binding to the LBD promotes receptor activation and transcriptional regulation in the nucleus

Collectively, endogenous PPARγ ligands (Fig. 2) can be viewed as lipid-derived immunometabolic signals generated during inflammation, oxidative stress, and metabolic flux. Their production within the CNS, particularly under neuroinflammatory conditions, supports the concept that PPARγ functions as a sensor of the local inflammatory and metabolic milieu.

### Synthetic Ligands: Pharmacological Activation and Selective Modulation - TZDs and Emerging Derivatives

Synthetic activation of PPARγ has been most prominently explored through TZDs, a class of compounds primarily represented by TGZ, CGZ, RGZ, lobeglitazone and PIO, which were developed as insulin-sensitizing agents for the treatment of type 2 diabetes mellitus [72–75]. Structurally, these ligands share a 2,4-thiazolidinedione scaffold containing two carbonyl groups and heteroatoms that provide a polar interaction region, while chemical modifications of the scaffold and its substituents allow substantial variation in receptor affinity, transcriptional efficacy, pharmacokinetics, and biological activity [76]. This structural optimization has shaped the clinical development of TZDs, with successive compounds aimed to retain the beneficial effects of full PPARγ agonism while improving their safety profiles, addressing severe idiosyncratic hepatotoxicity associated with TGZ, cardiovascular safety concerns associated with RGZ, and class-related adverse effects such as weight gain, edema, and fluid retention [77, 78].

**Fig. 3:**
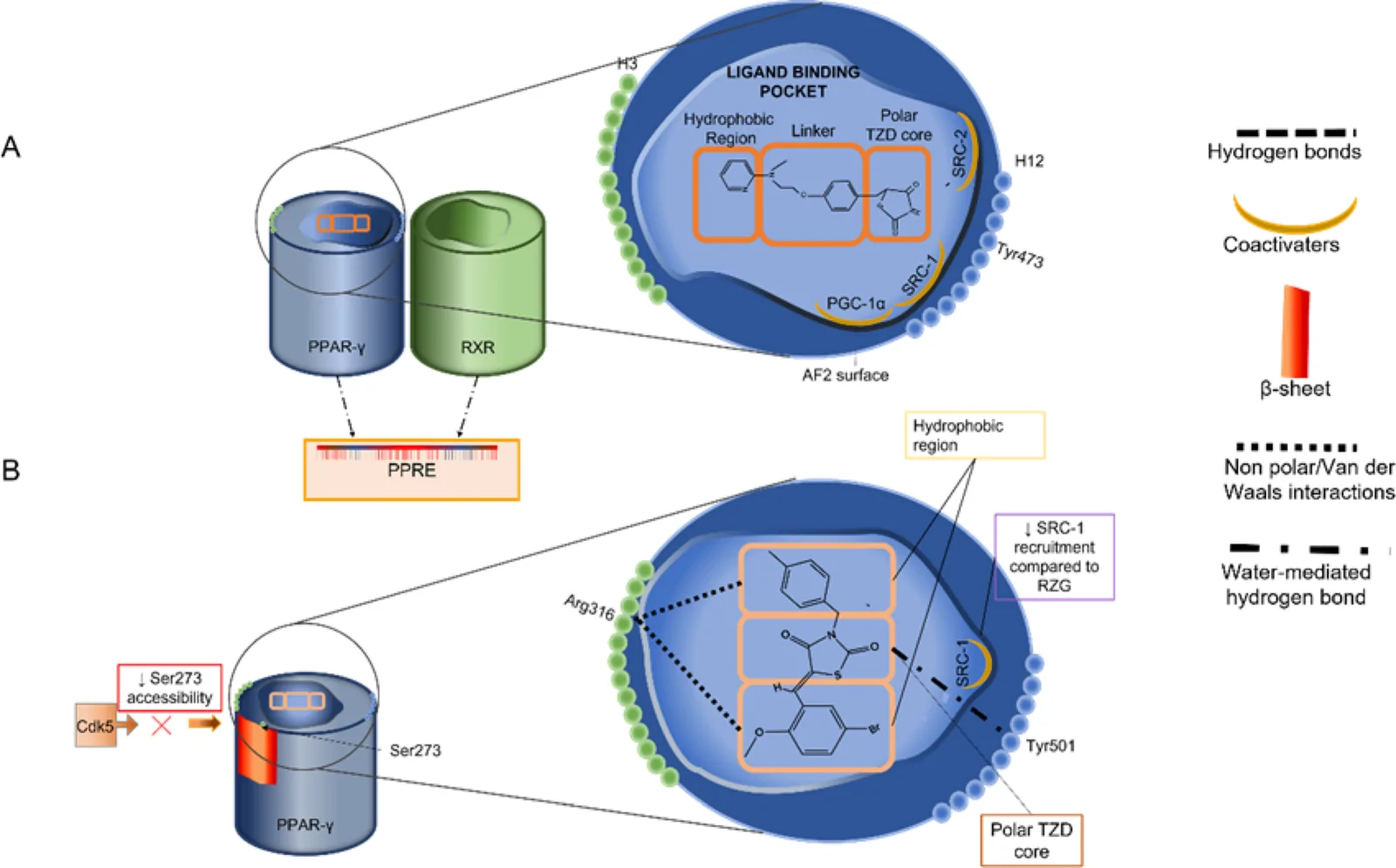
Structural and functional differences between full and partial PPARγ agonism. **A** RGZ exemplifies full agonism by strongly stabilizing H12 and the coactivator-competent AF-2 surface, thereby promoting broad coactivator recruitment and robust PPARγ-dependent transcription. **B** In contrast, GQ-16 preferentially stabilizes H3 and the β-sheet/Ser273 region, reducing Ser273 accessibility to Cdk5 and its subsequent phosphorylation while eliciting weaker classical coactivator-dependent transactivation

Classical full agonism and its transcriptional consequences are closely associated with the conformation of the LBD and the pattern of coregulator recruitment. In canonical full agonists such as RGZ, direct interactions with Helix 12 (H12) contribute to the stabilization of the AF-2 surface, favoring the recruitment of transcriptional coactivators, including SRC-1, SRC-2, and PGC-1α, and promoting robust PPARγ-dependent transcription [79]. However, maximal PPARγ transcriptional activation is not necessarily required to retain therapeutically relevant biological effects. Partial agonists can induce alternative conformational states of the LBD that favor selective regulation of metabolically beneficial PPARγ-responsive genes. As exemplified by GQ-16 in Fig. 3, stabilization of H3 and the β-sheet/Ser273 region reduces Cdk5-mediated Ser273 phosphorylation, thereby preserving selective PPARγ-regulated gene expression despite weak classical AF-2-dependent transactivation [80].

This structural redistribution of receptor stabilization has important biological consequences. Although GQ-16 is substantially less effective than classical TZDs in conventional transactivation assays and in promoting coactivator recruitment, it retains selected beneficial PPARγ-associated effects, including insulin-sensitizing adipokine responses, while inducing less adipogenesis [81, 82]. This provides mechanistic support for the development of SPPARMs, which also include balaglitazone, which retains antihyperglycemic efficacy while producing less fluid retention [83] PA-082 and GW0072 [79, 84], which show preferential PGC-1 recruitment relative to p160 coactivators, and MBX-102, which combines weak classical transactivation with efficient corepressor displacement and transrepression [85], collectively illustrating the mechanistic diversity of selective PPARγ modulation as summarized in Table 1.

**Table 1:** Representative synthetic PPARγ ligands and selective modulators: mechanistic profiles, pharmacological advantages, and major limitations

Ligand/pharmacological class	Mechanistic/transcriptional profile	Key pharmacological advantage	Principal limitation
TGZ, — classical TZD [86]	Classical PPARγ agonist, vitamin E-like chroman moiety may contribute antioxidant properties.	Insulin sensitization, first glitazone introduced clinically.	Severe hepatotoxicity associated with metabolic activation/reactive intermediates, withdrawn from clinical use.
PIO — full PPARγ agonist [87]	Classical full-agonist profile with strong H12/AF-2-dependent transcriptional activation.	Effective insulin sensitization and glycemic control, clinically established TZD.	Weight gain and fluid retention, class-related cardiovascular concerns.
RGZ — full PPARγ agonist [86, 87]	Prototypical full agonist, H-bonds with H323/H5, H449/H11 and Y473/H12 stabilize H12/AF-2.	Potent insulin sensitizer and reference full agonist for comparative PPARγ pharmacology.	Adipogenic and fluid-retentive effects, historical cardiovascular safety concerns.
Balaglitazone — selective partial agonist [83]	Reduced maximal PPARγ activation relative to RGZ, consistent with partial agonism.	Antihyperglycemic efficacy with less plasma-volume expansion and cardiac enlargement than RGZ.	Residual fluid-retentive/adipogenic effects, partial agonism attenuates rather than abolishes class liabilities.
GW0072 — partial agonist/SPPARM [88, 89]	Alternative LBD binding without direct H12 interaction, preferential PGC-1 recruitment over p160 coactivators.	Low adipogenic activity with selective coregulator recruitment.	Inhibits osteoblast differentiation/mineralization despite low adipogenic activity.
GQ-16 — TZD-derived selective partial agonist [80]	Binds parallel to H3 and stabilizes the H3/β-sheet/Ser273 region, reducing Cdk5-mediated Ser273 phosphorylation.	Preserves insulin sensitization with less adipogenesis, weight gain and edema.	Preclinical evidence, lower classical transactivation and reduced coactivator association.
PA-082 — non-TZD isoquinoline SPPARM/partial agonist [89]	Preferential PGC-1 recruitment over SRC-1/TIF2/SRC-3, no direct H12 interaction.	Supports insulin signaling/glucose uptake while limiting triglyceride accumulation and adipogenic responses.	Evidence is predominantly biochemical/cellular, in vivo validation was not established in the cited study.
MBX-102/JNJ39659100 (metaglidasen) — non-TZD SPPARM/partial agonist [85]	Weak classical transactivation with efficient NCoR/SMRT displacement and strong inflammatory transrepression.	Insulin sensitization and glucose lowering with minimal adipogenesis and reduced weight gain/edema in preclinical models.	Low receptor/transactivation potency, molecular basis of strong transrepression remains incompletely defined.
INT131 — non-TZD selective PPARγ modulator (SPPARM)/partial agonist [90]	Predominantly hydrophobic LBP binding with no direct Tyr473/H12 H-bond, selective coregulator recruitment and gene regulation rather than full-agonist-like AF-2 activation.	Reduced adipogenesis while preserving beneficial PPARγ signaling. In CNS models, ↑ PGC-1α, mitochondrial remodeling, dendritic complexity and survival against Aβ toxicity, also attenuated glial inflammatory markers and demonstrated brain penetration	CNS evidence remains preclinical, neural PPARγ occupancy and cell-specific coregulator recruitment were not measured, so the causal link between its LBD/coregulator bias and neuroprotection remains unresolved.
LSN862 — non-TZD PPARα/γ dual agonist, partial PPARγ agonist [91]	PPRE- and coregulator-dependent partial agonism: near-full ASC-2/C33 recruitment but reduced CBP, PGC-1 and TRAP220 recruitment versus RGZ, transcriptional efficacy varies across PPREs.	Crosses the BBB and produced dose-dependent preservation of nigrostriatal dopaminergic innervation, with reduced microglial/astrocytic activation and oxidative-stress responses in MPTP-treated mice.	Significant PPARα activity confounds PPARγ-specific attribution, treatment began before MPTP exposure, and neural coregulator engagement was not directly examined.

The pharmacological diversity of TZDs extends beyond canonical nuclear PPARγ signaling. PIO and related TZDs can interact with mitochondrial proteins such as mitoNEET/CISD1, suggesting that some effects on bioenergetics and redox homeostasis may occur independently of direct PPARγ-mediated transcription [92]. This is mechanistically relevant but also complicates interpretation, because mitochondrial effects observed with TZDs cannot automatically be attributed to PPARγ activation. Likewise, chemical modification of the TZD scaffold has generated compounds with PPARγ, anti-inflammatory, antimicrobial, or anticancer activities [87, 93].

The translational potential of selective PPARγ modulation may depend less on the magnitude of receptor activation than on the ligand-induced LBD conformational ensemble. NMR and HDX studies indicate that different ligands redistribute PPARγ among distinct conformational ensembles, which can subsequently modify coregulator affinity and transcriptional output [94]. Thus, ligand-specific conformational bias rather than reduced efficacy alone may provide the mechanistic basis for selective PPARγ pharmacology. The pharmacological diversity of TZDs extends beyond canonical nuclear PPARγ signaling. PIO and related TZDs can interact with mitochondrial proteins such as mitoNEET/CISD1, suggesting that some effects on bioenergetics and redox homeostasis may occur independently of direct PPARγ-mediated transcription [92]. This is mechanistically relevant but complicates interpretation, as mitochondrial effects of TZDs cannot automatically be attributed to PPARγ activation. Likewise, chemical modification of the TZD scaffold has generated compounds with PPARγ-modulatory, anti-inflammatory, antimicrobial, and anticancer activities [87, 93].

The translational potential of selective PPARγ modulation may depend less on the magnitude of receptor activation than on the ligand-induced LBD conformational ensemble. NMR and HDX studies indicate that pharmacologically distinct ligands shift PPARγ among different conformational states, thereby influencing coregulator affinity and transcriptional output [94]. Thus, ligand-specific conformational bias, rather than reduced efficacy alone, may provide a mechanistic basis for selective PPARγ pharmacology. Such conformational selectivity may be particularly relevant to CNS drug development, where effective ligands must combine appropriate receptor modulation with sufficient brain exposure, cellular distribution, and transcriptional activity in disease-relevant neural cell populations.

This concept is illustrated by the non-TZD ligands INT131 and LSN862. INT131, a SPPARM and partial PPARγ agonist, adopts a binding mode distinct from RGZ, lacks the characteristic direct Tyr473/H12 interaction, and produces a distinct coregulator-recruitment profile [90]. In CNS models, INT131 increased PGC-1α expression, altered mitochondrial dynamics, promoted neuronal survival, and attenuated glial inflammatory responses [95]. LSN862, a non-TZD PPARα/γ dual agonist with partial PPARγ activity, displays differential recruitment of ASC-2, C33, CBP, PGC-1, and TRAP220 and PPRE-dependent transcriptional efficacy. In the MPTP model, LSN862 crossed the BBB, reduced glial activation, and preserved nigrostriatal dopaminergic innervation [91], however, its concurrent PPARα activity precludes attribution of these effects exclusively to selective PPARγ modulation.

These observations suggest that CNS-oriented PPARγ pharmacology should move beyond a simple full-versus-partial agonist classification. This may be particularly important in the CNS, where neural and glial populations exhibit highly cell- and context-specific transcriptional programs and PPARγ activation can reshape microglial gene expression [56]. Future ligand development should therefore integrate LBP/conformational selectivity, coregulator recruitment, adequate brain exposure, PPAR-subtype and neural cell-type selectivity, and biomarkers of CNS target engagement, providing a more mechanistic basis for separating neuroprotective PPARγ signaling from the peripheral liabilities of classical TZDs.

## PPARγ as a Regulator of Synaptic Plasticity and Neuroimmune-Metabolic Homeostasis

Synaptic plasticity comprises activity-dependent modifications in the functional efficacy, molecular composition, and structural organization of synapses. In the hippocampus, long-term potentiation (LTP) involves partially distinct mechanisms of induction, expression, and maintenance, whose contributions vary according to the circuit and stimulation protocol employed [96–99]. Primarily, PPARγ is not part of the immediate machinery involved in LTP induction. Unlike N-methyl-D-aspartate receptors (NMDARs), Ca²⁺/calmodulin-dependent protein kinase II (CaMKII), and AMPA-type glutamate receptors (AMPARs) which trigger rapid synaptic changes, PPARγ functions as a delayed genomic regulator. Instead of driving acute ion channel flux or immediate receptor trafficking, PPARγ orchestrates downstream gene-expression programs required for LTP remodeling and metabolic adaptation, with its specific transcriptional output depending on cell-type cofactor availability and ligand-induced conformational changes [14, 100–102].

### Mitochondrial Bioenergetics and the Maintenance of Plasticity

The expression and maintenance of LTP impose a high energy demand on the neuroglial circuit. Adenosine triphosphate (ATP) is consumed by synaptic vesicle cycling, Na⁺/K⁺-ATPases and Ca²⁺-ATPases, intracellular transport, receptor trafficking, and local protein synthesis. At presynaptic terminals, vesicle mobilization, endocytosis, reacidification, and refilling also depend on ATP. In parallel, astrocytes use energy to remove glutamate and K⁺ from the extracellular space and regenerate glutamine. Plasticity is therefore not a metabolically autonomous response of the dendritic spine, but rather an emergent property of cooperation between neuronal and glial compartments [103–106].

Ca²⁺ influx through NMDARs and voltage-gated channels is partially coupled to mitochondrial Ca²⁺ uptake, which stimulates tricarboxylic acid cycle dehydrogenases and adjusts ATP production to synaptic activity. This coupling operates within a narrow functional range: mitochondrial uptake must support energy demand without progressing to matrix overload, loss of inner membrane potential, excessive ROS production, and opening of the mitochondrial permeability transition pore. Conversely, indiscriminate ROS suppression may interfere with physiological redox signals involved in kinase activation and glutamate receptor trafficking. Thus, mitochondrial function relevant to plasticity consists of coordinating ATP, Ca²⁺, and ROS according to the intensity and duration of synaptic activity, rather than independently maximizing or abolishing any of these parameters [107–111].

The functional interaction between PPARγ and PGC-1α recruitment provides a mechanism through which transcriptional programs may expand metabolic reserve. PGC-1α coactivates factors such as nuclear respiratory factor 1 (Nrf1), promoting the expression of respiratory-chain components and mitochondrial transcription factor A (TFAM), which participates in the organization, transcription, and replication of mitochondrial DNA. The enhancement of PGC-1α recruitment is also associated with mitochondrial and redox homeostatic pathways that include superoxide dismutase 2, catalase and uncoupling protein 2 (UCP2), although these proteins are not direct and universal targets of PPARγ in all CNS cell types [112, 113].

A functional link between mitochondrial capacity and synaptic remodeling was demonstrated by Cheng et al. [114]. In primary cultures of hippocampal neurons, PGC-1α overexpression increased mitochondrial content, spine density, and the molecular differentiation of synapses, whereas its silencing reduced spinogenesis and synaptogenesis. In vivo, reduced PGC-1α expression in mature dentate gyrus granule neurons also decreased spine density. Brain-derived neurotrophic factor (BDNF)-stimulated PGC-1α expression promoter activity through mechanisms involving ERK and CREB, and silencing the coactivator blocked BDNF-induced spine formation without preventing tropomyosin receptor kinase B (TrkB) activation. These findings demonstrate that PGC-1α participates in the conversion of neurotrophic signaling into structural remodeling [114]. However, because PPARγ was not manipulated, the study defines a potentially shared effector mechanism but does not provide evidence of direct receptor involvement.

This distinction is particularly important when interpreting studies involving thiazolidinediones. PGC-1α is also regulated by AMPK, sirtuin 1 (SIRT1), and BDNF-ERK-CREB, whereas PIO and RGZ may modify mitochondrial metabolism through mechanisms independent of PPARγ-mediated transcription, including inhibition of the mitochondrial pyruvate carrier [113, 115, 116]. Consequently, increases in PGC-1α, Nrf1, TFAM, ATP, or respiration following treatment with a thiazolidinedione do not prove that PPARγ caused synaptic recovery. Such an inference requires demonstrating receptor dependence and its association with functional outcomes, such as LTP or the synaptic localization of AMPARs.

### PPARγ–BDNF Signaling and Synaptic Remodeling

BDNF contributes to the late phase of LTP through the activation of TrkB and the PI3K-Akt-mTOR, Ras-ERK, and phospholipase C-γ pathways. These signaling pathways regulate CREB, dendritic translation, AMPAR trafficking, postsynaptic density protein 95 (PSD-95) organization, and actin cytoskeleton remodeling. Neurotrophic signaling, however, depends on the metabolic competence of the synapse: spine formation and stabilization require a coordinated expansion of mitochondrial capacity, as demonstrated by the dependence of BDNF-induced remodeling on PGC-1α [114, 117–120].

Kariharan et al. [121] provided the most direct experimental link between PPARγ, BDNF, and synaptic function. In hippocampal neuronal cells, the expression of a constitutively active form of PPARγ increased the activity of the promoter associated with exon IX of *Bdnf*, BDNF levels, glutamate receptor expression, and dendritic spine formation [121]. In db/db mice, intracerebroventricular administration of RGZ attenuated deficits in memory, post-tetanic potentiation, and LTP without restoring peripheral insulin sensitivity. Central administration reduces the likelihood that these effects can be explained exclusively by peripheral metabolic sensitization, whereas the promoter assay supports a transcriptional association between PPARγ and *Bdnf*.

However, the study design did not resolve the entire causal sequence. It was not demonstrated that AMPA or NMDA receptors were redistributed to the synaptic membrane or that increased BDNF was necessary for electrophysiological recovery, because BDNF or TrkB was not blocked during the recordings. Furthermore, neuronal *Pparg* deletion was not performed in vivo. Thus, the findings support a functional connection among PPARγ, BDNF, glutamatergic components, and plasticity, but do not establish BDNF as the exclusive mediator of LTP recovery [121].

The PI3K-Akt pathway represents a potential point of convergence among PPARγ, TrkB, and insulin signaling. Akt promotes mTOR-dependent translation and phosphorylates GSK3β at Ser9, thereby reducing its activity. Studies using metabolic and neurodegenerative models have associated PIO administration with improved insulin signaling, increased Akt, and inhibitory phosphorylation of GSK3β [122–124]. In their narrative review, Alqahtani et al. [125] placed the loss of PI3K/Akt signaling and GSK3β disinhibition at the interface among brain insulin resistance, synaptic dysfunction, and Alzheimer’s disease (AD) pathology. This convergence makes indirect modulation of this axis by PPARγ agonists biologically plausible, but does not demonstrate direct activation of PI3K by the receptor or establish PI3K-Akt-GSK3β as an obligatory mediator of its synaptic effects [125].

The association among TZDs, BDNF, and plasticity has also been investigated under endocrine-metabolic conditions. In rats with propylthiouracil-induced juvenile hypothyroidism, Hosseini et al. [126] observed, through in vivo electrophysiological recordings from the Schaffer collateral-CA1 pathway, that PIO and RGZ attenuated the reduction in field excitatory postsynaptic potential (fEPSP) amplitude and slope following high-frequency stimulation [126]. The improvement was accompanied by increased hippocampal BDNF, reduced NO₂⁻/NO₃⁻ metabolites, and lower TUNEL labeling. The coexistence of these outcomes associates synaptic recovery with reduced nitrosative stress and cellular preservation, but does not define a causal sequence. The study did not employ PPARγ antagonism or deletion or BDNF/TrkB blockade, additionally, RGZ increased serum T4 concentration, introducing a possible contribution from hormonal correction.

In 3xTg-AD mice, Steinke et al. [29] evaluated AU9, a ligand with agonist activity at PPARδ and partial agonist activity at PPARγ. Following in vivo treatment, recordings from hippocampal slices showed recovery of theta-burst stimulation-induced LTP at Schaffer collateral-CA1 synapses [29]. The electrophysiological response was accompanied by increased BDNF, NGF, and NT-3, recovery of spine density, and changes in synaptic proteins, including PSD-95, GluR1, and NMDAR1 [29]. The agreement among electrophysiological, molecular, and morphological outcomes provides more consistent functional evidence than the isolated measurement of neurotrophins or behavior. However, the dual activity of AU9 and the absence of antagonism or individual deletion of each receptor preclude attributing the recovery exclusively to PPARγ. The study supports a multi-PPAR strategy but does not establish the necessary or sufficient contribution of each isoform.

Taken together, these models address complementary levels of plasticity. Kariharan et al. [121] provided neuronal and transcriptional evidence related to PPARγ; Hosseini et al. [126] demonstrated a pharmacological association between thiazolidinediones and LTP under hormonal interference; and [29] demonstrated electrophysiological and structural recovery following dual PPARδ/γ modulation. This convergence supports the ability of PPARs to promote conditions compatible with synaptic maintenance, whereas differences in the models, pharmacological selectivity, and experimental design preclude the conclusion that neuronal PPARγ is universally required for LTP.

### PPARγ and Astroglial Glutamate Homeostasis

Glutamatergic transmission depends on the rapid removal of extracellular glutamate and its recycling through the glutamate–glutamine cycle. Astrocytes take up a large proportion of released glutamate through EAAT2/GLT-1 and EAAT1/GLAST, with EAAT2/GLT1 predominating in the adult forebrain. Within the astroglial compartment, glutamate can be converted into glutamine by glutamine synthetase and returned to neurons or converted into α-ketoglutarate and incorporated into the tricarboxylic acid cycle. Neurotransmitter clearance is therefore directly connected to astroglial energy metabolism [105, 127, 128].

Reduced astroglial uptake prolongs the presence of glutamate in the extracellular space, increases its spillover, and promotes the recruitment of extrasynaptic NMDARs. Excessive activation of these receptors may antagonize pro-plasticity programs associated with synaptic NMDARs and reduce CREB activity, thereby compromising synaptic maintenance [129–132].

Romera et al. [133] linked PPARγ to the regulation of EAAT2/GLT-1 in cortical neuron-astrocyte cocultures subjected to oxygen-glucose deprivation preconditioning. RGZ increased EAAT2/GLT-1 mRNA and protein levels, enhanced [³H]glutamate uptake, and increased by approximately fourfold the activity of a reporter construct containing a region of the EAAT2/GLT-1 promoter. The PPARγ antagonist T0070907 prevented the preconditioning-induced increases in transporter expression and glutamate uptake. In addition, GLT-1 silencing using an antisense oligonucleotide and its inhibition by dihydrokainic acid reduced the neuroprotection produced by preconditioning and RGZ [133].

These findings support the involvement of PPARγ in the functional regulation of EAAT2/GLT-1 and protection against excitotoxicity in a system containing astrocytes. However, the design did not allow the response to be attributed exclusively to astrocytic PPARγ, and the absence of PPRE mutagenesis or promoter occupancy assays prevented the demonstration of direct receptor binding to the EAAT2/GLT-1 gene. The study also did not assess extracellular glutamate during synaptic activity, extrasynaptic NMDARs, the AMPA/NMDA ratio, or LTP. Therefore, enhanced glutamate clearance represents a plausible mechanism through which PPARγ may preserve an environment permissive to plasticity, but this connection has not yet been directly demonstrated through electrophysiology and selective manipulation of Pparg in astrocytes.

### PPARγ and Microglial Regulation of the Synaptic Environment

Microglia modify circuit organization through contact with pre- and postsynaptic elements, the selective elimination of synapses, and the release of cytokines and trophic factors. The direction of these responses depends on the brain region, developmental stage, and intensity and duration of microglial activation. During development and experience-dependent adaptation, the selective removal of connections contributes to circuit refinement. Under pathological conditions, however, excessive complement activation or the loss of antiphagocytic signals, such as CD47–SIRPα, may promote aberrant synaptic elimination [134–137].

Microglial cytokines also exert concentration- and context-dependent effects. Physiological IL-1β and TNF-α signaling may participate in plasticity and homeostatic synaptic scaling, whereas their persistent elevation impairs LTP through mechanisms that include p38 MAPK, altered AMPAR trafficking, and interference with neurotrophic signaling [138–140]. Prieto et al. [141] demonstrated that IL-1β and TNF-α suppressed chemical potentiation in hippocampal synaptosomes, confirming a direct effect within the synaptic compartment, although this system does not identify microglia as the source of the cytokines. Consequently, indiscriminate microglial suppression is not necessarily equivalent to the restoration of plasticity, because it may reduce harmful mediators while simultaneously impairing circuit surveillance and remodeling functions [142, 143].

The involvement of PPARγ in the microglial inflammatory response has been directly examined in primary cultures and microglial cell lines. In lipopolysaccharide (LPS)-stimulated rat primary microglia-enriched cultures, Xing et al. [144] observed that PIO reduced inducible nitric oxide synthase (iNOS) expression and nitric oxide production, decreased p38 MAPK phosphorylation, and increased PI3K/Akt signaling. Because antagonism, silencing, or deletion of Pparg was not employed, these findings demonstrate a pharmacological response to PIO but do not establish receptor dependence [144].

In LPS-stimulated BV2 cells, Choi et al. [145] found that galangin, a flavonol with a potential PPARγ modulation, reduced iNOS, nitric oxide, TNF-α, and IL-6, increased IL-10, and modified the NF-κB, Nrf2, and CREB pathways. Pharmacological antagonism and PPARγ silencing attenuated these effects, providing more consistent evidence of receptor involvement, although galangin has multiple molecular targets [145]. Also in BV2 cells, Han et al. [146] demonstrated that 6-shogaol preserved IκBα, reduced p65 phosphorylation and nuclear translocation, and decreased TNF-α, IL-1β, IL-6, and PGE₂. The partial reversal of these responses by GW9662 indicates PPARγ involvement but does not exclude receptor-independent mechanisms.

In primary murine microglia exposed to LPS, Zhang et al. [147] reported that asperosaponin VI reduced pro-inflammatory mediators, increased anti-inflammatory mediators, and modified markers of PPARγ signaling, GW9662 attenuated some of these effects. Taken together, these studies support the involvement of PPARγ in limiting microglial inflammatory programs. However, the strength of the causal evidence is not uniform: only some experimental designs combined antagonism and silencing, and all employed compounds capable of acting on other targets.

More importantly, these experiments did not assess the phagocytosis of synaptic elements, microglia-spine contacts, postsynaptic currents, the AMPA/NMDA ratio, or LTP. They demonstrate modulation of the microglial inflammatory phenotype but do not establish that this modulation is sufficient to restore plasticity. Establishing causality will require selective manipulation of Pparg in microglia combined with the quantification of inflammatory mediators, synaptic elimination, and electrophysiological function within the same model.

### Mechanistic Integration and Strength of the Evidence

The available evidence supports a model in which PPARγ regulates the conditions required for the sustained expression and maintenance of plasticity rather than acting as a component of canonical LTP induction. In the neuronal compartment, PPARγ has been associated with *Bdnf* transcription, glutamate receptor expression, spine formation, and LTP recovery [121]. In systems containing astrocytes, its pharmacological activation increased the expression of glutamate transporter GLT-1/EAAT2 and enhanced glutamate uptake, although the specific contribution of the astrocytic receptor has not yet been isolated [133]. In microglia, PPARγ-dependent or partially dependent interventions reduced inflammatory programs associated with NF-κB, p38 MAPK, and iNOS, but their direct effects on synapses and LTP remain unknown [139, 144].

These responses may converge on ATP availability, glutamate homeostasis, Ca²⁺ buffering, redox signaling, ERK–CREB activation, AMPAR trafficking, and spine stability. However, this convergence does not imply a linear cascade controlled exclusively by PPARγ [105, 148, 149]. PGC-1α, BDNF, TrkB, PI3K/Akt, and glutamate transporters are regulated by multiple pathways, and the relative contribution of each component varies among neurons, astrocytes, and microglia [144].

The strength of the conclusions should reflect the experimental design. Promoter assays support transcriptional regulation; antagonists and silencing provide evidence of receptor dependence, with limitations related to selectivity [150]; conditional deletions establish necessity in defined cell populations [151, 152]; and the combination of molecular markers with electrophysiology and synaptic morphology provides greater functional relevance [153, 154]. In this context, Kariharan et al. [121] provide the most direct connection among PPARγ, Bdnf, glutamatergic components, spines, and LTP. Cheng et al. [114] demonstrate the requirement for PGC-1α in BDNF-induced structural remodeling but do not test PPARγ. Hosseini et al. [126] provide a pharmacological association under hormonal interference, whereas Steinke et al. [29] demonstrate synaptic recovery using a dual ligand that does not allow specific attribution to PPARγ. Romera et al. [133] and the microglial studies define plausible glial mechanisms but do not directly connect them to LTP.

Addressing these gaps requires conditional deletion of PPARγ in neurons, astrocytes, and microglia, combined with fEPSP and patch-clamp recordings, AMPA/NMDA ratio measurements, paired-pulse ratio, synaptic localization of AMPARs, CaMKII and CREB phosphorylation, spine analysis, and measurements of ATP, mitochondrial respiration, ROS, and Ca²⁺ [109, 114]. In astrocytes, these approaches should be combined with the quantification of EAAT2/GLT-1 and extracellular glutamate [133]; in microglia, they should be combined with analyses of phagocytosis and synaptic elimination [134, 137]. This design will make it possible to determine whether PPARγ is required for the maintenance of plasticity or whether it acts primarily by increasing synaptic resilience to metabolic, excitotoxic, and inflammatory stress [114, 133].

## PPARγ in Neuroinflammation and Neurodegenerative Disorders

The contribution of PPARγ to neurodegeneration varies according to the primary disease mechanism and the affected cell populations [155]. In AD, its modulation has been associated with APP/Aβ processing, tau hyperphosphorylation and synaptic localization, and the glial response [29, 156]. In Parkinson’s disease (PD), studies have focused on dopaminergic vulnerability, mitochondrial dysfunction, neuroinflammation, and α-synuclein [157, 158]. In X-linked adrenoleukodystrophy (X-ALD), PPARγ has been investigated in the context of lipotoxicity, oxidative stress, and axonal degeneration resulting from ABCD1 deficiency [159, 160]. This evidence should be interpreted according to whether receptor involvement was directly demonstrated or merely pharmacologically inferred.

The heterogeneity of cellular responses is a central aspect of this interpretation [161]. Shokr [38] emphasized that microglial activation in neurodegenerative diseases is accompanied by the reprogramming of glycolysis, oxidative phosphorylation, and fatty acid oxidation, regulated by sensors such as HIF-1α, AMPK, and mTOR. PPARγ may promote lipid oxidative metabolic programs and restrict components of the inflammatory response [38, 56]. However, the microglial states observed in vivo are dynamic and do not correspond to a rigid binary classification; they vary according to the disease, brain region, pathological stage, and interactions with other nervous system cells [161, 162]. Consequently, modification of a single marker does not demonstrate a functionally neuroprotective microglial response.

### AD: Proteotoxicity, Metabolism, and the Glial Response

AD is characterized by progressive cognitive decline associated with extracellular deposition of amyloid-β (Aβ) peptides, and intracellular accumulation of hyperphosphorylated tau protein, forming neurofibrillary tangles [163–167]. These changes coexist with cerebral glucose hypometabolism, mitochondrial dysfunction, and persistent microglial and astrocytic responses [168–170]. These pathological hallmarks are accompanied by synaptic loss, and sustained neuroinflammation, all of which contribute to neuronal vulnerability and network disintegration [171–175].

Aging—the primary risk factor for AD—is associated with chronic low-grade neuroinflammation, marked by microglial priming, increased production of pro-inflammatory cytokines (IL-1β, IL-6, TNF-α), chemokines such as MCP-1, and complement activation [176–179]. Activated microglia surrounding Aβ plaques amplify oxidative stress and inflammatory cascades, exacerbating neuronal dysfunction and impairing synaptic plasticity [180–183].

Plaque-associated microglia exhibit alterations in phagocytosis and lipid metabolism, including lipid-droplet accumulation associated with reduced Aβ uptake [184]. Fibrillar Aβ can activate pattern-recognition receptors, such as TLR2 and TLR4, inducing inflammatory mediators and promoting activation of the NLRP3-caspase-1 inflammasome and IL-1β maturation [185–187]. In primary microglia, pharmacological activation of PPARγ increased Aβ uptake in a receptor- and CD36-dependent manner; in the APP/PS1 model, this response was accompanied by increased microglial Aβ content and reduced amyloid burden [188]. However, studies showing only reductions in glial markers or Aβ burden do not, in isolation, allow the conclusion that the inflammatory state was resolved or that peptide phagocytosis and degradation were increased [189].

In APPV717I mice, Heneka et al. [189] observed that short-term PIO treatment reduced glial activation and the cerebral concentration of soluble Aβ1–42. The response, which was partially similar to that produced by ibuprofen, reinforced the involvement of inflammatory mechanisms sensitive to PPARγ activation. However, the study did not quantify microglial Aβ internalization, its endolysosomal trafficking, or intracellular degradation. Therefore, the reduction in peptide levels cannot be directly interpreted as increased microglial phagocytosis. Direct repression of BACE1 was also not demonstrated, which would require analysis of promoter activity, occupancy of regulatory regions, and loss of the effect following PPARγ blockade or deletion.

Badhwar et al. [190] used mass spectrometry to analyze the proteome of cerebral arteries isolated from transgenic mice overexpressing APP. Among the 193 proteins altered in the model, PIO normalized 60, including HSPA5/GRP78, an endoplasmic reticulum chaperone associated with proteostasis that has been described as an APP-interacting protein. This normalization is consistent with the reorganization of vascular protein homeostasis but does not demonstrate direct regulation of HSPA5 by PPARγ, repression of BACE1, or a specific reduction in the amyloidogenic cleavage of APP. Furthermore, the reduction in APP following treatment only approached statistical significance (*p* = 0.07). Thus, the study mainly supports the partial recovery of the cerebrovascular proteome altered by APP overexpression, whereas its causal relationship with Aβ generation remains indirect [190].

Peripheral effects constitute another source of ambiguity. In Tg2576 mice, Pedersen et al. [191] demonstrated that RGZ improved spatial learning and memory, reduced serum corticosterone, attenuated the reduction in cerebral insulin-degrading enzyme expression and activity, and reduced cerebral Aβ42 levels without modifying amyloid deposition. Administration of metyrapone, an inhibitor of glucocorticoid synthesis, reproduced the reduction in corticosterone and the cognitive improvement observed with RGZ, supporting the functional involvement of the endocrine axis. Because prolonged glucocorticoid exposure may impair hippocampal plasticity and function, part of the cognitive benefit may have resulted from the correction of peripheral endocrine alterations [191–193]. However, because the study did not quantify free RGZ in the brain, PPARγ occupancy, or neuron- and glia-specific transcriptional responses, its findings do not allow the benefit to be attributed exclusively to cerebral receptor activation.

Brain insulin resistance represents an additional source of heterogeneity in AD. In a pharmacological review, Alqahtani et al. [125] linked alterations in metabolic signaling to the progression of Aβ and tau pathologies, neuroinflammation, and synaptic dysfunction. Because this analysis was not based on experimental manipulation of Pparg, it broadens the metabolic context in which its ligands may act but does not demonstrate a causal contribution of the receptor. This distinction is translationally relevant because patients with different degrees of insulin resistance, vascular disease, and metabolic impairment may respond differently to PPARγ modulation.

Models of scopolamine-induced cognitive impairment provide evidence regarding the cholinergic and oxidative components of cognitive vulnerability but do not fully reproduce the neuropathology of AD. In aged rats treated with scopolamine, Beheshti et al. [194] demonstrated that PIO improved performance in the Morris water maze and passive avoidance test. Treatment also reduced malondialdehyde and increased thiol-group content and superoxide dismutase and catalase activities in the hippocampus and cortex, although the magnitude of the responses varied according to dose and brain region [194]. These findings associate cognitive improvement with attenuation of oxidative imbalance in aged animals but do not demonstrate modification of Aβ or tau pathology.

In mice, Xiang et al. [195] found that PIO attenuated scopolamine-induced deficits in the Morris water maze and passive avoidance test. Behavioral improvement was accompanied by the preservation of acetylcholine and choline acetyltransferase activity and reduced acetylcholinesterase activity in the hippocampus or cortex, linking the pharmacological effect to the maintenance of cholinergic neurotransmission [195]. However, because scopolamine produces acute muscarinic antagonism, this paradigm predominantly assesses symptomatic procognitive activity and does not reproduce progressive Aβ deposition, tauopathy, or chronic neurodegeneration. Therefore, the findings do not demonstrate that PIO modifies the progression of AD.

In a complementary model of natural aging, Zhang et al. [196] administered α-lipoic acid to 18-month-old C57BL/6 mice and observed improved performance in the T-maze, reduced microgliosis, astrogliosis, and pro-inflammatory cytokines in the hippocampus, increased PPARγ expression, and reduced NF-κB phosphorylation. In BV2 cells subjected to D-galactose-induced senescence, the PPARγ antagonist GW9662 abolished the anti-inflammatory response to α-lipoic acid, supporting receptor involvement in this component of the effect [196]. However, PPARγ was not blocked in the animals during behavioral assessment; therefore, the study does not demonstrate that the in vivo cognitive improvement depended exclusively on the receptor. Moreover, the model reproduces aging and neuroinflammation but not the progressive Aβ and tau pathologies characteristic of AD.

The experimental evidence in AD supports three levels of response that should not be regarded as equivalent: modulation of the glial response, alteration of Aβ production or clearance, and structural or functional preservation of synapses. Markers such as IBA1 and GFAP, Aβ, HSPA5, neurotrophins, spine density, and LTP represent distinct compartments of the pathological cascade. Thus, reduced glial immunoreactivity does not demonstrate functional resolution of inflammation; reduced Aβ does not ensure synaptic recovery; and improvements in LTP or behavior do not, in isolation, demonstrate lasting modification of neuropathology [29, 189, 190].

Taken together, these findings illustrate a recurring divergence: classical PPARγ agonists such as PIO and RGZ reliably reduce amyloidogenic processing and neuroinflammation in preclinical models, yet this benefit does not extend to newer, brain-optimized agents’ full clinical potential being realized, nor to human trials using the same classical compounds [197, 198]. The most plausible explanation is limited CNS penetration: classical TZDs cross the blood–brain barrier poorly at clinically tolerated doses, whereas AU9 achieved superior brain bioavailability and correspondingly outperformed classical agonists in reducing Aβ burden [29]. This suggests that the therapeutic ceiling of PPARγ activation in AD is set less by mechanistic validity than by pharmacokinetic access to the target tissue.

### PD: Dependence on the Model and Therapeutic Timing

PD is characterized by progressive degeneration of dopaminergic neurons in the substantia nigra pars compacta (SNPDpc), resulting in striatal dopamine depletion and motor symptoms including bradykinesia, rigidity, tremor, and postural instability [199–204]. Non-motor symptoms—depression, cognitive impairment, and sleep disturbances—reflect broader network dysfunction beyond the nigrostriatal pathway [205].

PD pathophysiology involves the loss of nigrostriatal innervation, and reduced dopamine availability in the striatum, accompanied by α-synuclein-associated alterations in proteostasis. Mitochondrial and lysosomal dysfunction, oxidative stress, impaired autophagy/mitophagy, and neuroinflammatory responses interact in dopaminergic vulnerability [206–209]. However, the investigation of PPARγ in this context is predominantly based on neurotoxic models that reproduce specific components of nigrostriatal injury but not the slow and multifactorial progression of the human disease.

MPTP, MPP⁺, rotenone, and 6-hydroxydopamine produce lesions through distinct mechanisms, at different rates, and with different anatomical distributions. These models allow the assessment of tyrosine hydroxylase (TH) immunoreactivity, striatal dopamine content, motor performance, and glial responses, but incompletely reproduce the progressive aggregation and propagation of α-synuclein [210, 211]. In addition, because MPTP requires metabolic conversion into the active neurotoxic metabolite MPP⁺, outcomes obtained in this model may be influenced by the specific pharmacokinetic and toxicological properties of the experimental protocol. These features should be considered when interpreting apparent neuroprotective effects [210, 211].

Kumar et al. [212] studied rats subjected to bilateral MPTP infusion into the substantia nigra pars compacta. PIO, administered beginning five days before the lesion and maintained for 30 days, improved performance in the passive avoidance test and the cued version of the Morris water maze, reduced malondialdehyde, and partially restored glutathione [212]. Because cytokines, glial activation, and NF-κB components were not evaluated, the study supports an association between cognitive improvement and attenuation of oxidative stress, but not an anti-inflammatory effect. In another preventive protocol, Barbiero et al. [213] compared the PPARγ agonist PIO with the PPARα agonist fenofibrate in rats subjected to intranigral MPTP administration. Both preserved locomotor activity, performance in the two-way active avoidance test, TH immunoreactivity, striatal dopamine and its metabolites, and reduced caspase-3 activation [213]. However, treatment initiated five days before MPTP assessed resistance to acute injury rather than recovery after established neurodegeneration; furthermore, the absence of receptor antagonism or deletion precludes attributing all effects exclusively to PPARγ or PPARα.

The influence of therapeutic timing was directly examined by Barbiero et al. [214]. A dose of PIO administered 1 h after MPTP partially restored locomotor activity and striatal dopamine content, whereas daily administration maintained for 22 days did not produce equivalent neurochemical protection. However, neither regimen reversed the deficit observed in the two-way active avoidance test [214]. These findings indicate that efficacy on dopaminergic markers was greater when the intervention occurred immediately after the neurotoxin, but they do not demonstrate cognitive recovery or protection after established degeneration. The temporal dependence observed in these models limits their translation to patients, in whom nigrostriatal loss is already substantially established at the time of diagnosis.

Scuderi et al. [158] investigated Aesculus hippocastanum extract in SH-SY5Y cells exposed to MPP⁺. Treatment increased cell viability and restored tyrosine hydroxylase (TH) expression, while reducing α-synuclein immunopositivity, NF-κB activation, the release of IL-1β, IL-17, and TNF-α, and indicators of oxidative and nitrosative stress [158]. PPARG silencing attenuated the protective effects of the extract, providing evidence of receptor dependence in this experimental system. However, this inference remains restricted to an acute cellular model: SH-SY5Y cells do not fully reproduce the phenotype of mature dopaminergic neurons, MPP⁺ does not model the progression of synucleinopathy, and the multicomponent composition of the extract prevents identification of the ligand responsible for PPARγ activation. Thus, the study demonstrates functional receptor involvement in the cellular response to the extract but does not establish efficacy in animal or clinical models of PD [158].

Combined strategies have also been examined in neurotoxic models of PD. Das et al. [215] treated MPTP-lesioned rats with the PPARγ agonist GW1929 combined with α-lipoic acid, a pleiotropic compound associated with the modulation of PGC-1α and redox homeostasis. The combination, used at doses lower than those employed in the monotherapies, attenuated cognitive deficits and oxidative and inflammatory alterations, in parallel with the recovery of mitochondrial biogenesis markers [215]. These findings suggest pharmacological cooperation between PPARγ activation and PGC-1α-regulated programs. However, the absence of PPARγ antagonism or deletion and the multiple actions of α-lipoic acid preclude attributing the effects exclusively to the PPARγ-PGC-1α axis or establishing the causal order among mitochondrial recovery, reduced inflammation, and cognitive improvement [215].

A more indirect association was reported by Elkady et al. [157] in BALB/c mice exposed to rotenone. The combination of canagliflozin with levodopa/carbidopa attenuated behavioral alterations, striatal dopamine depletion, oxidative imbalance, and mitochondrial dysfunction more markedly than the monotherapies, accompanied by modulation of Nrf2 and the SIRT1/PPARγ and RAGE/HMGB1/NF-κB axes. However, canagliflozin is not an established PPARγ agonist, and the study did not assess receptor binding, PPRE transactivation, or PPARγ loss of function. Therefore, the alteration in tissue PPARγ content should be interpreted as a pharmacodynamic association within a multitarget response rather than as a demonstration that the receptor mediated the observed neuroprotection [157].

The collective interpretation of studies in PD should distinguish among the preservation of TH, recovery of striatal dopamine, motor improvement, and modification of α-synuclein. These outcomes represent different levels of protection. The maintenance of TH-positive cells in a toxic model does not demonstrate lasting functional preservation of the nigrostriatal circuit, just as motor improvement does not demonstrate modification of α-synuclein aggregation or propagation.

This contrast is instructive: pretreatment with PIO before MPTP exposure robustly prevented dopaminergic loss, whereas chronic treatment initiated after injury failed to reverse cognitive deficits. The difference is therapeutic timing relative to disease stage — once neurodegenerative cascades are established; anti-inflammatory and mitochondrial support alone cannot reverse the accumulated network-level damage. This same principle likely underlies the heterogeneous outcomes across PD patient populations in clinical trials, where disease duration and severity at enrollment varied substantially [216].

### PPARγ in X-ALD: Bioenergetics, Axonopathy, and Inflammation

X-ALD represents a paradigmatic model of metabolic neurodegeneration, in which ABCD1 transporter deficiency impairs peroxisomal β-oxidation of very-long-chain fatty acids (VLCFAs). This deficit leads to progressive accumulation of these neurotoxic lipids, triggering a cascade of cellular events including mitochondrial dysfunction, oxidative stress, and activation of neuroimmune responses, particularly in vulnerable brain regions [217, 218]. The dynamic nature of X-ALD distinguishes it from simple lipid storage disorders: VLCFA-induced lipotoxicity acts as a trigger for self-amplifying circuits that perpetuate axonal degeneration, oligodendrocyte loss, and immune cell infiltration, contributing to the rapid clinical progression observed in cerebral forms of the disease [219, 220].

Cellular models have elucidated the central inflammatory mechanisms underlying this pathology [160, 221]. VLCFA accumulation persistently activates the NF-κB pathway, promoting transcription of pro-inflammatory cytokines such as TNF-α, IL-1β, and IL-6, as well as enzymes including COX-2, while amplifying inflammatory lipid mediators [219, 221, 222]. This coordinated activation perpetuates microglial activation, triggering a chronic inflammatory cycle that results in oxidative stress, mitochondrial damage, and oligodendrocyte death [219, 221, 222]. The consequence of this pro-inflammatory environment is the consolidation of lesion niches that promote progressive axonal degeneration and functional impairment, directly reflecting the neurological manifestations of the disease [219, 223].

PPARγ agonists capable of penetrating the CNS emerge as promising modulators of these processes [224]. In vitro studies show that leriglitazone restores mitochondrial bioenergetics in neurons and astrocytes exposed to VLCFA toxicity, increasing intracellular ATP levels and reducing oxidative stress markers [160, 225]. Moreover, leriglitazone modulates the cellular inflammatory phenotype by attenuating NF-κB activation and reducing expression of pro-inflammatory cytokines and enzymes, suggesting that PPARγ activation directly interferes with central circuits of lipotoxicity-induced degeneration [160]. These effects reflect not only bioenergetic protection but also structural and functional preservation of neurons and astrocytes, indicating potential therapeutic relevance in the context of X-ALD [120].

Furthermore, in ABCD1-deficient murine models, PPARγ activation with leriglitazone restores mitochondrial function parameters in nervous tissue, reduces oxidative stress, and decreases glial reactivity, halting axonal degeneration and promoting measurable functional improvement [160]. Complementary studies using PIO demonstrated correction of mitochondrial deficits, normalization of the NAD+/NADH ratio, reduction of oxidative damage, and preservation of locomotor function in Abcd1 -/- mice [226]. These results indicate that different PPARγ agonists act on multiple physiological levels, from cellular bioenergetics to preservation of axonal integrity, reinforcing the translational potential of modulating this pathway in the CNS [160, 226].

### Clinical Studies in Neurodegenerative Disorders

Clinical trials with PIO and RGZ did not reproduce the neuroprotective effects observed in experimental models of AD and PD. In NET-PD FS-ZONE, 210 participants with early PD, concomitantly treated with rasagiline or selegiline, were randomized to receive PIO at doses of 15 or 45 mg or placebo for 44 weeks. The mean change in the total Unified Parkinson’s Disease Rating Scale score was 4.42 points with 15 mg, 5.13 with 45 mg, and 6.25 with placebo. Although numerically lower than placebo, the results for both doses met the prespecified futility criteria, indicating a low probability of sufficient benefit to justify a confirmatory trial [216].

NET-PD FS-ZONE did not quantify PIO in cerebrospinal fluid, PPARγ occupancy, transactivation of PPRE-containing genes, or changes in central inflammatory markers [216]. Therefore, the study demonstrated a lack of disease-modifying efficacy under the evaluated regimen but did not allow determination of whether the failure resulted from insufficient brain exposure, lack of receptor engagement, or irreversibility of the nigrostriatal lesion. The inclusion of patients at an early stage partially reduced the problem of therapeutic timing, but “early PD” still represents a stage subsequent to the loss of a substantial proportion of dopaminergic terminals [216].

In mild-to-moderate AD, Gold et al. [227] randomized 693 participants, stratified by APOE ε4 genotype, to placebo, extended-release RGZ at doses of 2 or 8 mg, or donepezil as an active control for 24 weeks. Neither dose of RGZ improved the coprimary ADAS-Cog and CIBIC+ outcomes, either in the overall population or in the stratum without APOE ε4. Peripheral edema occurred in 15% of participants who received 8 mg and in 5% of those who received placebo. The lack of response in the stratum without APOE ε4 weakened the previously suggested pharmacogenetic signal but does not demonstrate that APOE is irrelevant; it indicates that its use alone did not identify a population responsive to RGZ [227].

Harrington et al. [228] reported two phase 3 studies in which extended-release RGZ, at doses of 2 or 8 mg, was added to stable treatment with acetylcholinesterase inhibitors in patients with mild-to-moderate AD. The intervention did not produce significant improvements in cognition or global clinical assessment, including in analyses stratified by APOE ε4. These findings indicate that the failure of RGZ was not overcome by maintaining symptomatic cholinergic treatment. However, the studies continued to select participants based on clinical diagnosis without systematic confirmation of Aβ, tau, brain insulin resistance, or central PPARγ engagement [228].

Geldmacher et al. [197] evaluated PIO titrated to 45 mg over 18 months in 29 nondiabetic participants with probable AD, 25 of whom completed the study. The trial was designed primarily to evaluate safety and was not statistically powered to establish clinical efficacy. No significant differences were identified in exploratory analyses of cognition, activities of daily living, neuropsychiatric symptoms, or global function. Peripheral edema occurred in 28.6% of treated participants and in none of the placebo participants, but no withdrawals were attributed to adverse events. The study demonstrates the absence of an exploratory efficacy signal and the occurrence of an adverse effect characteristic of the drug class but does not constitute definitive evidence of PIO inefficacy [197].

TOMMORROW moved the intervention to a cognitively asymptomatic stage but did not resolve the limitations related to dose, engagement, and biological selection. A total of 3,494 participants were enrolled; 3,061 were classified as being at high risk according to an APOE- and TOMM40-based algorithm, of whom 1,545 were randomized to extended-release PIO at 0.8 mg/day and 1,516 to placebo. Mild cognitive impairment associated with AD occurred in 39 of 1,430 analyzed participants in the PIO group and in 46 of 1,406 in the placebo group, with no significant difference. The study was terminated after failing to meet the prespecified nonfutility threshold [198].

The low dose used in TOMMORROW was selected to reduce peripheral effects, but brain exposure or PPARγ transactivation in the CNS was not demonstrated. In addition, genetic risk classification did not require confirmation using biomarkers of Aβ, tau, or neurodegeneration. Thus, the study included participants with different probabilities of developing cognitive impairment due to AD and possibly with distinct pathological mechanisms. The result demonstrates that advancing the timing of intervention and using genetic stratification are insufficient when molecular pathology and target engagement remain unconfirmed [198].

X-ALD provides a relevant translational contrast because it has a defined primary metabolic alteration and was investigated using an agonist developed to reach the CNS. In ADVANCE, 116 men with adrenomyeloneuropathy were randomized in a 2:1 ratio to leriglitazone or placebo for 96 weeks. The dose was adjusted to achieve a prespecified plasma exposure, but leriglitazone did not improve the primary outcome, defined as the change in distance covered in the six-minute walk test. Therefore, the study did not demonstrate reduced functional progression of myelopathy [229].

The secondary findings of ADVANCE should be interpreted separately from the primary outcome. Clinical progression to cerebral X-ALD occurred in six of the 39 participants who received placebo and in none of the 77 participants treated with leriglitazone. Plasma MMP-9 decreased with treatment, whereas increased neurofilament light chain (NfL) was observed mainly among placebo participants who exhibited progression of cerebral lesions; therefore, it should not be stated that leriglitazone globally reduced NfL. These findings support a hypothesis of activity on the neurovascular or cerebral component but remain secondary and do not negate the negative primary functional outcome [229].

In NEXUS, García-Cazorla et al. [230] evaluated leriglitazone in boys aged 2–12 years with cerebral lesions in an open-label phase 2/3 study planned for 96 weeks. In the 24-week interim analysis, the 11 evaluable participants exhibited slowed lesion growth or stabilization, and five met the criterion for arrested progression. The participants remained clinically stable, and neurological, Loes, and gadolinium enhancement intensity scores stabilized in most cases. However, the absence of a concurrent control group, the small number of participants, and the comparison with natural history preclude attributing the observed course exclusively to leriglitazone [230].

The comparison across diseases indicates that the presence of a defined metabolic alteration improves biological selection but does not eliminate dependence on phenotype and outcome [229, 230]. In X-ALD, the absence of benefit for myelopathy in ADVANCE coexisted with exploratory signals related to cerebral progression [229], whereas NEXUS directly evaluated children with cerebral lesions [230]. Therefore, adrenomyeloneuropathy and cerebral X-ALD should not be treated as a single therapeutic population [229–231]. Likewise, the preliminary NEXUS findings cannot be directly compared with negative controlled trials in AD and PD without considering differences in study design, sample size, and the molecular definition of the disease [198, 216, 228, 230].

The gap between experimental neuroprotection and clinical efficacy results from a combination of uncertain brain exposure to classical thiazolidinediones, the absence of biomarkers of PPARγ occupancy or transactivation, intervention after substantial structural damage, molecular heterogeneity of the populations, and peripheral effects that restrict the dose [198, 216, 232, 233]. Relatively homogeneous toxic or transgenic models also reproduce only components of human diseases, whereas experimental outcomes frequently measure resistance to an acute injury rather than modification of a progressive pathology [198, 216] Future trials should sequentially demonstrate CNS exposure, PPARγ engagement, modification of the pathological mechanism, and functional preservation in biomarker-selected populations [234, 235]. Improvement in TH, Aβ, LTP, or behavior in an experimental model does not, in isolation, constitute evidence of clinical disease modification [198, 216].

## Pharmacological Implications and Translational Priorities

The development of PPARγ modulators for neurological diseases should distinguish four properties that are not necessarily equivalent: receptor affinity, brain exposure, cofactor recruitment pattern, and functional response. SPPARMs stabilize receptor conformations distinct from those induced by full agonists and may consequently produce specific profiles of coregulator recruitment and gene expression. However, molecular selectivity does not ensure cellular selectivity: because the availability of coactivators and corepressors varies among neurons, astrocytes, microglia, and vascular cells, the same ligand may induce different responses according to the cell population and pathological stage [90, 224, 232].

Dual or pan-PPAR agonists represent another possibility for diseases characterized by simultaneous metabolic and inflammatory alterations. The combined activation of PPARα, PPARδ, and PPARγ may broaden the regulation of lipid oxidation, mitochondrial capacity, and inflammatory transcription, but it also complicates the attribution of effects to a specific isoform and may increase systemic actions. AU9 exemplifies this limitation: in 3xTg-AD mice, the compound, characterized as a PPARδ agonist and partial PPARγ agonist, improved cognition, LTP, spine density, and neuropathological markers. These findings support possible cooperation between PPARδ and PPARγ but do not demonstrate the required contribution of each receptor [29]. Future studies should combine ligands with defined pharmacological profiles, selective antagonism, conditional deletions, and cell type-specific transcriptomic analyses.

Selectivity for PPARγ should also be distinguished from functional convergence among neuroprotective pathways. Badawi et al. [236] demonstrated that clemastine attenuated seizures, cognitive impairment, oxidative alterations, and inflammatory responses in rats subjected to pentylenetetrazole kindling, in association with sigma-1 receptor modulation. Clemastine was not characterized as a PPARγ ligand in this study, and the findings do not constitute direct evidence of the involvement of this receptor. Its contribution to the present discussion is comparative: pharmacologically distinct targets may converge on oxidative stress, mitochondrial function, and inflammation [236]. This convergence provides a rationale for investigating combination therapies, but its validation requires the independent manipulation of each target and demonstration that the combination produces additional benefit without increasing toxicity.

Increasing CNS exposure represents an additional priority. The low brain exposure of classical thiazolidinediones has been proposed as a possible explanation for the discrepancy between their preclinical and clinical effects, whereas leriglitazone was specifically developed as a PPARγ agonist with increased brain penetration [224]. Nanocarriers, prodrugs, intranasal formulations, and ligands with favorable physicochemical properties should be investigated as potential strategies rather than regarded as already validated solutions for PPARγ modulators. Furthermore, the total brain concentration is not equivalent to the free concentration available for receptor interaction because part of the compound may remain bound to proteins, membranes, or tissue lipids. Pharmacokinetic studies should therefore quantify free exposure and relate it to target engagement and the pharmacodynamic response [237, 238].

Clinical planning should incorporate an explicit sequence of exposure, target engagement, and biological response. Quantification of the free drug in plasma and, when feasible, cerebrospinal fluid may demonstrate whether the administered dose produces exposure compatible with central activity. PPARγ engagement should be assessed using transcriptional or protein signatures previously validated for the ligand and tissue analyzed, because no cerebrospinal fluid biomarker is currently established as a specific measure of brain occupancy by this receptor [229, 239, 240]. Changes in adiponectin may provide exploratory information on systemic PPARγ activity but do not, in isolation, demonstrate brain engagement. Brain injury biomarkers, such as NfL and GFAP, together with markers of microglial activation and inflammatory remodeling, such as TREM2 and MMP-9, may be incorporated into response or injury panels, but they reflect distinct processes and should not be treated as substitutes for PPARγ activation or clinical benefit [229, 239, 240].

Participant stratification should extend beyond syndromic diagnosis and consider the predominant biological mechanism. In AD, confirmation of amyloidosis and tauopathy, combined with assessment of the extent of neurodegeneration and vascular comorbidities, may reduce the heterogeneity of experimental groups [234]. In PD, biomarkers of pathological α-synuclein, dopaminergic integrity, and clinical progression may help define more biologically homogeneous populations, although their application as predictors of therapeutic response still requires validation [234]. In X-ALD, adrenomyeloneuropathy and inflammatory cerebral disease should be analyzed separately because they exhibit different immediate mechanisms, rates of progression, and clinical outcomes [229, 230]. The therapeutic window should be defined by measurable biological states rather than solely by the time elapsed since diagnosis.

PPARγ therefore emerges as a context-dependent pharmacological target whose relevance varies according to the primary disease mechanism, cell population, ligand profile, and pathological stage [241–243]. Clinical translation will require compounds with demonstrated brain exposure, characterization of cofactor recruitment, specific engagement biomarkers, and outcomes related to the mechanism of each disease [14, 238, 240]. This approach will make it possible to distinguish pharmacological exposure, PPARγ modulation, experimental neuroprotection, and effective clinical modification, which are levels of evidence that should not be regarded as equivalent [198, 216].

## PPARγ in Psychiatric Disorders

The involvement of PPARγ in psychiatric disorders varies according to diagnosis, neural circuit, cell population, and metabolic and inflammatory status [244–246]. In anxiety disorders, the most consistent evidence links the receptor to the modulation of stress vulnerability through central and peripheral mechanisms [244, 245]. In depression, the findings depend on the predominant component of each model, including chronic stress, immune activation, and metabolic dysfunction [247–249]. Anxiety disorders, major depressive disorders and bipolar depression should therefore be analyzed separately because the available studies differ in the pathophysiology investigated, the population selected, and the consistency of the clinical findings [244, 250–252].

### Anxiety Disorders

Anxiety disorders comprise heterogeneous conditions characterized by excessive threat appraisal, hypervigilance, persistent anticipation of adverse events, and difficulty regulating emotional responses. Neurobiological studies implicate distributed circuits that include the amygdala, prefrontal cortex, and hippocampus; however, no single neural signature is shared across all diagnoses, and the direction of functional alterations depends on the disorder, population investigated, clinical state, and experimental paradigm employed [253–255].

In this context, research on PPARγ remains predominantly preclinical and has focused on the modulation of stress responses, as demonstrated by neuronal manipulation of the receptor in the amygdala and by studies evaluating stress-associated prefrontal and amygdalar oscillations in mice, rather than on models that fully reproduce specific psychiatric diagnoses [244, 256]. This distinction is fundamental because baseline anxiety, adaptive defensive responses to threats, and persistent anxiety-like behaviors are not equivalent phenomena. Consequently, changes in avoidance, exploration, or emotional reactivity following experimental stress may identify mechanisms related to susceptibility or resilience but, in isolation, do not demonstrate therapeutic efficacy against an established anxiety disorder [257–259].

#### Preclinical Evidence: Central and Peripheral Regulation of Stress Vulnerability

Experimental evidence indicates that PPARγ may regulate stress vulnerability through central and peripheral mechanisms that are not mutually exclusive. Domi et al. [244] demonstrated a central contribution in mice with neuronal deletion of PPARγ. Loss of the receptor produced limited alterations in baseline anxiety but intensified the anxiety-like response following acute stress, meanwhile restoration of PPARγ in the amygdala normalized this vulnerability. In the same study, PIO at 30 mg/kg prevented the anxiogenic effect of stress without substantially affecting unstressed animals, whereas GW9662 blocked this protection [244]. The combination of neuronal deletion, regional rescue, agonism, and antagonism demonstrates that PPARγ is causally involved in stress adaptation, with a function more closely related to circuit resilience than to constitutive suppression of anxiety.

Guo et al. [245] identified a peripheral contribution in mice subjected to chronic social defeat stress. Susceptible, but not resilient, animals exhibited reduced PPARγ, particularly the PPARγ2 isoform, and adiponectin in adipose tissue, and the levels of these components correlated with social interaction. RGZ increased adiponectin production and attenuated behavioral alterations related to anxiety and depression, whereas evidence obtained in adiponectin-deficient animals confirmed the importance of this adipokine in the stress response and the observed behavioral effects [245, 260]. Comparison with Domi et al. [244] indicates that stress-related behavioral phenotypes may result from both neuronal regulation of PPARγ in the amygdala and peripheral endocrine–metabolic signaling. Consequently, the systemic effects of TZDs cannot be attributed exclusively to cerebral PPARγ occupancy.

Circuit dependence becomes evident when these findings are compared with the GABAergic results. Vanaveski et al. [261] observed in vitro that PIO and GW1929 increased expression of the α2 subunit of GABA_A_ receptors in primary neurons. In vivo, mice with neuronal overexpression of PGC-1α exhibited increased GABA_A_ α2 expression and altered hippocampal GABAergic transmission, but also greater anxiety-like behavior [261]. This finding does not directly contradict Domi et al. (2016), because PGC-1α overexpression is not equivalent to selective PPARγ activation and because the manipulations emphasized distinct regions. Instead, it demonstrates that an increase in one component of inhibitory transmission does not predict the direction of behavior, which depends on the neuronal population, receptor localization, and network organization.

Stress duration also modifies the response. Whereas Domi et al. (2016) evaluated an acute challenge and Guo et al. (2017) used repeated social defeat, Yuan et al. (2024) subjected mice to seven weeks of chronic restraint stress [244, 245, 249]. Luteolin increased exploration of the central area in the open-field test without changing the total distance traveled, indicating reduced anxiety-like behavior without a nonspecific locomotor effect [249]. The compound also attenuated depressive-like phenotypes and modified the microglial response, and GW9662 abolished these changes [249]. Because anxiety, depression, and inflammation were evaluated jointly, the study cannot determine whether the improvement in anxiety resulted from a specific action on threat circuits or from a general reduction in the consequences of stress [249]. Collectively, these models support PPARγ as a contextual regulator of stress vulnerability, but not as a uniform anxiolytic mechanism.

#### Clinical Evidence and Translational Gaps in Anxiety Disorders

The causal evidence obtained in animals has not yet been reproduced in patients with anxiety disorders. In EPICAMP, Roohafza et al. [262] randomized 104 nondiabetic individuals with metabolic syndrome to receive PIO, titrated to 30 mg/day, or placebo for 24 weeks. Although anxiety scores decreased over the course of the study, this reduction was comparable between groups and did not demonstrate a specific anxiolytic effect of PIO [262]. Moreover, participants were selected based on the presence of metabolic syndrome rather than a primary anxiety diagnosis, precluding extrapolation of the findings to generalized anxiety disorder or panic disorder [262].

Cosgrove et al. [263] provided limited human evidence linking PPARγ to neural processing relevant to emotional regulation. In a double-blind, placebo-controlled, repeated-measures pharmacological fMRI study, 20 healthy participants received placebo, 200 mg ibuprofen, or 600 mg ibuprofen before an emotional face-matching task [263]. Higher peripheral PPARγ expression was associated with greater activation of the insula and transverse temporal and superior temporal gyri, whereas increasing ibuprofen doses were associated with reduced BOLD activation in the dorsolateral prefrontal cortex and fusiform gyrus [263]. Importantly, ibuprofen neither significantly altered PPARγ expression nor interacted with receptor expression, indicating that the observed association between peripheral PPARγ expression and neural responses to emotional stimuli was not attributable to pharmacological modulation of PPARγ [263]. Because the study involved healthy participants and did not assess anxiety symptoms or therapeutic response, these findings support a potential relationship between PPARγ-related biology and emotional processing but do not establish a direct anxiolytic effect [263].

### Major Depressive Disorder

Major depressive disorder (MDD) is a heterogeneous syndrome in which anhedonia, cognitive alterations, sleep disturbances, psychomotor symptoms, and somatic manifestations occur in variable combinations, forming partially distinct symptom profiles within the same diagnostic category [264]. This heterogeneity also extends to pathophysiology: only a subset of patients exhibits persistent inflammation, insulin resistance, or marked metabolic alterations, whereas other phenotypes are more strongly associated with hypothalamic–pituitary–adrenal axis dysregulation, stress exposure, and different neurobiological and environmental alterations [265–267].

Studies of inflammatory and immunometabolic depression therefore support the existence of biologically distinct subgroups within the diagnosis of MDD rather than a pathological mechanism shared by all patients [265–269]. PPARγ has greater plausibility as a target in phenotypes with a demonstrable immunometabolic component, but this association does not justify characterizing MDD as a whole as an inflammatory or metabolic disease.

#### Preclinical Evidence: Dependence on the Model and Pathological Component

Experimental models reproduce different dimensions of depression and must be compared according to the predominant pathological stimulus, duration of exposure, and outcomes assessed. In an acute pharmacological study in mice, Rosa et al. [270] primarily investigated NP031115, a TZD with activity at GSK-3β and PPARγ. Both NP031115 and RGZ, administered intracerebroventricularly, reduced immobility in the forced swim test, and GW9662 prevented these effects. The absence of a relevant locomotor alteration indicated that reduced immobility did not simply result from nonspecific motor stimulation [270]. The study therefore identified a central response that was pharmacologically dependent on PPARγ, but it neither established genetic causality nor determined the responsible cell population. Furthermore, the acute forced swim test assesses a coping strategy in response to an immediate stressor and does not reproduce persistent anhedonia, prolonged stress exposure, or the clinical heterogeneity of MDD [270].

In a prolonged model, Zhao et al. [246] demonstrated that PIO attenuated the reduction in sucrose preference and increase in immobility induced by chronic mild stress in mice. Treatment also reduced hippocampal expression of pro-inflammatory mediators, including IL-1β, IL-6, TNF-α, and iNOS, and modified the microglial activation profile by decreasing markers associated with the pro-inflammatory response and increasing components related to the alternative response. Complementary experiments in LPS-treated N9 microglial cells showed that PIO increased IκBα and reduced NF-κB activity [246]. GW9662 attenuated the behavioral, molecular, and cellular effects of PIO, supporting PPARγ involvement, although systemic pharmacological antagonism did not identify the indispensable cell population or exclude peripheral actions [246]. The transition from an acute behavioral assay to a prolonged model strengthens the relationship among PPARγ, stress adaptation, and microglial regulation, but does not demonstrate that neuroinflammation is required in all depressive phenotypes [246].

Comparison with an inflammatory model shows that convergent behavioral outcomes may emerge from distinct pathological conditions. Liao et al. [248] administered LPS intracerebroventricularly and observed that pretreatment with PIO attenuated depressive-like behaviors. This response was accompanied by inhibition of the NF-κB–IL-6–STAT3 axis, restoration of CREB–BDNF signaling, and modification of proteins associated with serotonergic neurotransmission. GW9662 attenuated the molecular and behavioral effects of PIO, providing pharmacological evidence of PPARγ involvement [248].

Unlike the chronic stress paradigm used by Zhao et al., however, the model employed by Liao et al. produced acute and intense central immune activation [246, 248]. Agreement between the studies supports greater relevance of PPARγ when inflammation contributes to the experimental phenotype, but it does not establish neuroinflammation as a universal mechanism of MDD. Nor does it demonstrate that restoration of CREB–BDNF necessarily mediated the behavioral improvement, because these alterations were evaluated concomitantly and were not subjected to specific causal blockade [246].

The metabolic contribution was more evident in the study by Kurhe and Mahesh [247], who combined diet-induced obesity with chronic unpredictable mild stress. In this context, PIO restored sucrose preference, reduced immobility, and improved metabolic and neuroendocrine alterations associated with obesity and stress, including insulin resistance and increased corticosterone [247]. The study demonstrates that metabolic status may amplify or modify behavioral phenotypes and the response to PPARγ activation; however, because PIO also improved systemic parameters, the behavioral benefit cannot be attributed exclusively to cerebral receptor occupancy [247].

Guo et al. provided additional evidence for this peripheral communication. In mice subjected to chronic social defeat, susceptible, but not resilient, animals exhibited reduced PPARγ, particularly the PPARγ2 isoform, and adiponectin in adipose tissue [245]. The levels of these components correlated with social interaction, whereas RGZ increased adiponectin expression and circulating concentration and attenuated behavioral alterations related to anxiety and depression. The functional involvement of adiponectin was supported by genetic and pharmacological manipulations, showing that the adipose PPARγ–adiponectin axis contributes to stress susceptibility [245, 260].

Collectively, the studies by Kurhe and Mahesh and Guo et al. show that the effects of TZDs may involve communication between adipose tissue and the brain, particularly in the presence of obesity, insulin resistance, or adipose dysfunction [245, 247]. Nevertheless, behavioral responses were also observed in acute, chronic stress, and central LPS administration models without previously established metabolic dysfunction. Insulin resistance and inflammation may therefore define subgroups with greater plausibility for PPARγ modulation, but they are not universal requirements for the receptor’s preclinical effects.

#### Clinical Evidence in MDD

Clinical findings suggest potential antidepressant activity of TZDs, but do not simply relate the benefit to improved insulin resistance. Rasgon et al. [271] added RGZ to the treatment of 12 patients with unipolar or bipolar depression and insulin resistance for 12 weeks; eight completed the study. Among completers, Hamilton Depression Rating Scale scores decreased by approximately 39%, and four exhibited a reduction of more than 50% in severity. Insulin sensitivity improved, but its change did not correlate with symptom reduction [271].

Kemp et al. [272] evaluated PIO for 12 weeks in patients with MDD and abdominal obesity or metabolic syndrome and observed a reduction in Inventory of Depressive Symptomatology scores from approximately 40.3 to 19.2 points, accompanied by decreases in HOMA-IR and high-sensitivity C-reactive protein [272]. Among partial responders, the improvement was maintained during an optional extension of up to 24 weeks [272]. These studies provided concurrent clinical and metabolic signals, but their open-label designs did not allow the pharmacological effect to be distinguished from regression to the mean, expectations, and concomitant treatment [272].

Sepanjnia et al. [250] tested the hypothesis in a randomized, double-blind, placebo-controlled trial involving 40 patients with moderate-to-severe MDD [250]. Twenty received citalopram combined with PIO at 15 mg twice daily, and 20 received citalopram combined with placebo for six weeks [250]. At week 6, clinical response occurred in 95% of participants treated with PIO and 40% of those receiving placebo; remission occurred in 45% and 15%, respectively. The finding demonstrates a short-term adjunctive benefit, but not efficacy as monotherapy, maintenance of response, or long-term safety [250]. In EPICAMP, Roohafza et al. [262] evaluated 104 individuals with metabolic syndrome over 24 weeks and observed a greater reduction in depression scores with PIO (*p* = 0.011; time-by-treatment interaction, *p* = 0.032), without superiority for anxiety or stress. The change in depression was not significantly correlated with HOMA-IR (*p* = 0.145), indicating that the mood response was not directly explained by improved insulin resistance [262].

Taken together, the open-label studies by Rasgon et al. [271] and Kemp et al. [272] provided exploratory signals in metabolically compromised populations; Sepanjnia et al. [250] demonstrated an adjunctive benefit in patients with MDD without requiring metabolic syndrome; and Roohafza et al. [262] observed improvement in depressive symptoms in participants selected specifically for metabolic alterations. Positive findings in metabolically distinct populations weaken the use of insulin resistance alone as a predictor of response. At the same time, the small samples, concomitant treatments, and absence of measurements of brain exposure and target engagement preclude establishing whether the effects were mediated by PPARγ in the CNS [250, 262, 271, 272].

### Bipolar Depression: Divergent Clinical Evidence

Bipolar depression should be analyzed separately from MDD because it has a distinct clinical course, background treatments, and therapeutic risks. Kemp et al. [273] administered adjunctive PIO for eight weeks to 34 patients with bipolar depression and metabolic syndrome or insulin resistance. Depressive symptoms and some cardiometabolic parameters improved, with a more pronounced mood response among participants with greater baseline insulin resistance [273]. The absence of a placebo group, however, prevents the effect of PIO from being distinguished from the course of the episode and concomitant treatment [273]. Zeinoddini et al. [251] tested PIO at 30 mg/day or placebo as an adjunct to lithium for six weeks in patients with bipolar I disorder without diabetes or metabolic syndrome. The time-by-treatment interaction was significant for Hamilton Depression Rating Scale scores (*p* = 0.005), and score reductions were greater with PIO at weeks 2, 4, and 6 [251]. This finding indicates that the positive response was not restricted to metabolically compromised patients.

In contrast, Aftab et al. [252] found no antidepressant superiority of PIO in an eight-week randomized, double-blind, placebo-controlled trial. Insulin resistance improved in the treated group, but the metabolic change did not translate into a mood benefit [252]. The divergence between Zeinoddini et al. [251] and Aftab et al. [252] cannot be resolved by metabolic status: the positive trial excluded diabetes and metabolic syndrome, whereas the negative trial documented improved insulin resistance without a corresponding psychiatric response. Differences in samples, background treatments, and statistical power may have contributed, but neither study demonstrated brain exposure, PPARγ occupancy, or target-gene transactivation [252]. Positive findings do not prove central mediation by the receptor, and negative findings do not determine whether failure resulted from lack of target engagement, clinical heterogeneity, or ineffectiveness of the mechanism [252].

### Translational Barriers and Research Priorities

The gap between experimental models and human disorders constitutes a fundamental translational barrier. Animals subjected to LPS, chronic stress, or social defeat form relatively homogeneous populations exposed to known stimuli over controlled periods, whereas patients have distinct clinical trajectories, comorbidities, environmental exposures, previous treatments, and metabolic and inflammatory states [274, 275]. Moreover, each experimental paradigm reproduces only specific dimensions of psychopathology, such as anhedonia, social avoidance, or passive stress coping, without fully recapitulating the clinical heterogeneity of depressive and anxiety disorders [274, 275].

The timing of intervention also differs: in several animal studies, compounds were administered before or during development of the phenotype, as in models of chronic stress, social defeat, and immune activation, whereas clinical trial participants already had established affective episodes [245, 246, 248, 251, 252, 273]. Preventing behavioral alterations during stress exposure is therefore not equivalent to reversing persistent adaptations in emotional circuits.

PIO and RGZ exert systemic effects on insulin sensitivity, adipose tissue, adiponectin, peripheral inflammation, and vascular physiology, making it difficult to attribute behavioral alterations exclusively to cerebral PPARγ [245, 271, 273]. Although PIO reaches the brain in experimental models, its penetration is limited by pharmacokinetic properties and efflux transporters, including P-glycoprotein, and the exposure observed in animals does not demonstrate that clinical doses produce sufficient brain target engagement in psychiatric patients [233, 276].

None of the available psychiatric trials adequately quantified the unbound drug fraction in the brain, PPARγ occupancy, or central transactivation of genes containing PPAR response elements as pharmacodynamic measures of target engagement [250–252, 262, 271, 273]. Clinical benefit, metabolic improvement, brain exposure, and central receptor activation should therefore remain distinct levels of evidence. Edema, fluid retention, and weight gain are recognized adverse effects of TZDs and may restrict their long-term use, particularly in populations at cardiometabolic risk [277, 278]. Moreover, the six- to twelve-week duration of most psychiatric trials does not allow the persistence of benefit or its relationship to long-term safety to be adequately established.

Translational priorities should be specific to each diagnosis. In anxiety disorders, an initial clinical signal must be established in patients selected based on a primary diagnosis, because the available human studies evaluated symptoms in metabolic populations or emotional processing in healthy participants rather than therapeutic efficacy in established anxiety disorders [262, 263]. In MDD, new trials should prospectively assess whether insulin resistance, adiponectin, peripheral inflammation, or combinations of these markers identify responsive subgroups.

This approach is consistent with evidence that persistent inflammation characterizes only a subset of patients and that stratification according to immunometabolic phenotypes may reduce heterogeneity in antidepressant trials [266]. In bipolar depression, the discrepancy between the positive trial by Zeinoddini et al. (2015) and the negative result reported by Aftab et al. (2019) requires replication in larger samples, with standardized background treatments, metabolic stratification, and demonstration of pharmacological target engagement before confirmatory studies [251, 252]. In experimental models, conditional deletion of Pparg in neurons, microglia, and astrocytes should be combined with molecular, circuit-level, and behavioral measurements within the same design, allowing central cell-specific effects to be distinguished from peripheral metabolic consequences [151, 244, 246].

Collectively, PPARγ has causal evidence supporting its role in the regulation of stress vulnerability in anxiety models and a growing body of preclinical evidence in depressive phenotypes with microglial, inflammatory, or metabolic components [55, 244, 245, 249]. The clinical evidence, however, remains heterogeneous: small trials suggest a possible adjunctive benefit in MDD and in some metabolically selected populations [250, 262, 271, 272], whereas controlled findings in bipolar depression are divergent [251, 252]. For primary anxiety disorders, controlled evidence remains insufficient to establish clinical efficacy. This distribution positions PPARγ as a target potentially dependent on diagnosis, biological phenotype, and pharmacological target engagement, rather than as a uniform mechanism or validated treatment for emotional disorders as a whole [250, 262, 271, 272].

## PPARγ in Neuro-Oncology: Metabolic Reprogramming, Inflammation, and Tumor Plasticity

Although PPARs were originally characterized as regulators of lipid metabolism and glucose homeostasis, accumulating evidence indicates that these nuclear receptors also influence tumor biology, including brain neoplasms [279–281]. In the context of neuro-oncology, PPARγ has attracted particular attention due to its ability to modulate cellular metabolism, inflammation, differentiation, and apoptosis [279, 282].

Several studies report that PPARγ expression levels in primary brain tumors correlate with clinical outcomes, suggesting a context-dependent role in tumor progression [280, 283, 284]. In some settings, PPARγ activation induces differentiation and suppresses proliferation, supporting a tumor-suppressive function [283]. Mechanistically, PPARγ activation upregulates the tumor suppressor PTEN, thereby inhibiting PI3K/AKT signaling—a central pathway promoting cell survival, migration, and proliferation [283, 285, 286]. Through this axis, PPARγ reduces phosphorylation-dependent activation of AKT and downstream proliferative cascades. Furthermore, PPARγ activation inhibits epithelial–mesenchymal transition (EMT), a process that enhances tumor invasiveness and metastatic potential [283]. By preserving epithelial characteristics and limiting cytoskeletal remodeling, PPARγ reduces migratory capacity and invasion, thereby attenuating tumor aggressiveness [283, 285, 286].

Neuroblastoma is an embryonic neoplasm arising from neural crest-derived cells, representing the most prevalent extracranial solid tumor in childhood [287–289]. The PPARγ has been investigated as a key regulatory factor in this context, although its role appears to be highly dependent on the tumor subtype and the specific ligand employed [287–289].

Mechanistically, the selective inhibition of PPARγ in *MYCN*-amplified (v-myc myelocytomatosis oncogene) neuroblastoma cells (such as the CHP212 line) has demonstrated promising results in reversing the malignant phenotype. Nakao-Ise et al. [289] observed that treatment with the antagonist GW9662 induces cell cycle arrest at the G1 phase and promotes apoptosis in a dose-dependent manner. Notably, this antagonist suppresses the expression of proteins crucial for tumor survival and proliferation, including NMYC, BCL2, and BRD4. Beyond growth inhibition, PPARγ blockade induced neuronal differentiation, evidenced by morphological changes and the upregulation of the marker TUBB3 (beta-3 tubulin) in both in vitro assays and xenograft models [289].

TZDs and related agonists exert anti-proliferative effects in brain tumor cell lines through multiple mechanisms [290, 291]. The use of agonists such as RGZ presents varied effects depending on the experimental environment. Krieger-Hinck et al. [287] analyzed the impact of RGZ in SCID mice injected with SK-N-SH cells. Although RGZ significantly decreased the cell proliferation rate in primary tumors—dropping from approximately 57% in controls to 45–47% in treated groups—other clinical parameters were not substantially altered [287]. The findings indicated that primary tumor weight, apoptosis rates, and the development of pulmonary metastases were not considerably influenced by the agonist treatment in vivo [287].

The limitations observed with traditional agonists are further contextualized by the findings of Nakao-Ise et al. [289], who highlight the importance of the *MYCN* status in determining the response to PPARγ modulation. These results underscore that the direction of PPARγ modulation, whether through agonism or antagonism, must be carefully calibrated against the genetic pattern of the tumor to successfully trigger a shift from a proliferative to a differentiated state [289].

Bridging these observations, Jang et al. [288] provide a critical mechanistic advancement by investigating the repositioning of leflunomide as a potent inducer of neuroblastoma differentiation. The study reveals that leflunomide functions as a PPARγ agonist that uniquely upregulates the PGC-1α. This PPARγ/PGC-1α signaling axis was shown to be essential for the maturation of SH-SY5Y and SK-N-BE(2)C cells, resulting in robust neurite outgrowth and the expression of markers such as MAP2 and neurofilaments [288]. By integrating the observations of Krieger-Hinck et al. [287] and Nakao-Ise et al. [289], it becomes evident that the efficacy of PPARγ-targeted therapies relies not only on receptor activation but on the recruitment of specific coactivators like PGC-1α to overcome the differentiation block inherent in malignant neuroblastoma.

This contrast is instructive: RGZ, a classical full agonist, reduced proliferation only modestly and left apoptosis and metastatic spread unchanged [287], whereas leflunomide, acting through the same receptor, drove robust neuronal differentiation via PGC-1α recruitment [288].

Another highly aggressive brain tumor is glioblastoma (GBM). While neuroblastoma is an embryonic tumor of the neural crest, glioblastoma is an astrocytic glial tumor characterized by exceptionally low survival rates, a high degree of cellular heterogeneity, metabolic and mitochondrial dysfunction [282, 292–295]. Despite these distinct cellular origins, both malignancies share a critical dependence on survival pathways involving PPARγ signaling. As a lipid-sensitive transcription factor, PPARγ directly regulates genes involved in fatty acid uptake, lipid storage, and mitochondrial oxidative pathways [296]. Consequently, the activation of PPARγ may interfere with the specific metabolic adaptations required for tumor growth, offering a mechanistic parallel to the differentiation and homeostatic roles observed in neuroblastoma [292].

Recent studies have significantly updated the understanding of PPARγ signaling in glioblastoma, shifting the focus toward its role in tumor microenvironment modulation and the regulation of specific oncogenic drivers [297–300]. Contemporary evidence demonstrated that Ginsenoside RK3 exerts potent anti-glioblastoma effects by modulating macrophage M2 polarization through the PPARγ/CCL2 axis [298]. In this model, Ginsenoside RK3 acts as a PPARγ agonist, which in turn inhibits the recruitment and polarization of tumor-associated macrophages into the pro-tumorigenic M2 phenotype by suppressing CCL2 secretion [298]. This intervention reduced tumor volume in orthotopic xenograft models and inhibited the migration and neurosphere formation of U87 and GL261 cells, reinforcing the idea that PPARγ is a critical mediator in reversing the immunosuppressive glioblastoma microenvironment [298].

Further expanding the molecular network of PPARγ, Luo et al. [297] identified a regulatory link between the piggyBac transposable element derived 5 (PGBD5) and the PPAR signaling pathway. The study found that PGBD5 is highly expressed in glioma tissues and correlates with poor clinical prognosis. Mechanistically, the knockdown of PGBD5 was shown to upregulate the PPAR signaling pathway, leading to a marked inhibition of cell migration and invasion, alongside an induction of apoptosis and G0/G1 phase arrest in U251 and U87 cells [297] This suppression of malignant progression was validated in vivo, where PGBD5-silenced tumors exhibited slower growth rates and reduced weight, suggesting that the restoration of PPARγ activity is a fundamental consequence of targeting specific oncogenic transposases [297].

Complementing these protein-based mechanisms, the role of non-coding RNAs as indirect modulators of PPARγ has gained traction. Ordóñez-Rubiano et al. [301] underscored the therapeutic potential of miRNA-based approaches, noting that several miRNAs target metabolic and signaling nodes that intersect with the PPARγ pathway. Although the primary focus of microRNA research often involves direct silencing of oncogenes like E2F3a or the modulation of the PI3K/AKT axis, the integration of these miRNAs into the broader metabolic framework of glioblastoma suggests that they may function as upstream regulators of PPARγ activity [301]. By influencing the metabolic reprogramming, specifically glycolysis and lipid metabolism, these miRNA-mediated changes can sensitize glioma cells to PPARγ-dependent growth inhibition and differentiation [301]. Together, these findings transition the narrative from simple receptor activation to a multi-layered regulatory strategy involving epigenetic silencing, immune modulation, and microRNA-driven metabolic control.

Glioblastomas exist within a highly immunologically active microenvironment characterized by tumor-associated macrophages, activated microglia, and elevated cytokine production [299, 300]. Chronic NF-κB activation within tumor and stromal cells promotes angiogenesis, invasion, and resistance to apoptosis. Activation of PPARγ suppresses NF-κB signaling, reduces the expression of pro-inflammatory cytokines, and promotes the reprogramming of macrophage polarization, thereby modulating the inflammatory microenvironment and influencing tumor progression in a context-dependent manner [302, 303]. Moreover, PPARγ influences angiogenesis by modulating VEGF signaling and endothelial cell behavior. While PPARα and PPARβ/δ are often associated with anti-angiogenic effects, PPARγ’s impact appears context-dependent, potentially contributing to either suppression or modulation of tumor vascularization depending on ligand type and tumor stage [19].

A striking feature of PPARγ biology in neuro-oncology is that both agonism and antagonism have independently produced antitumor effects, depending on context — a pattern better explained by four converging factors than by any single mechanism. First, tumor subtype: the paradox is sharpest in neuroblastoma, where PPARγ antagonism (GW9662) induced differentiation and apoptosis in MYCN-amplified cells [289], while PPARγ agonism (leflunomide) induced comparable differentiation in other neuroblastoma lines via PGC-1α recruitment [288]— opposite pharmacological strategies converging on the same phenotypic endpoint within the same tumor type. In glioblastoma, by contrast, the evidence reviewed here points almost exclusively toward agonism-associated benefit; no comparable antagonist effect has been reported, which may reflect that GBM has simply been tested less extensively with PPARγ antagonists rather than a genuine absence of antagonist efficacy in this tumor type — a gap that future studies should address directly. Table 2 summarizes how these four factors diverge between the two tumor types discussed above.

**Table 2:** Factors underlying the divergent, context-dependent effects of PPARγ agonism and antagonism in neuroblastoma and glioblastoma

Factor	Neuroblastoma	Glioblastoma	Key evidence
Cell of origin/PPARγ-responsive target	Neural crest-derived neuronal precursor cells; PPARγ acts tumor-cell-intrinsically, driving terminal neuronal differentiation of the malignant cells themselves	Astrocytic glial lineage, within a highly heterogeneous and immunologically active microenvironment; PPARγ’s antitumor effects are documented mainly through reprogramming of surrounding immune cells rather than direct differentiation of tumor cells	Nakao-Ise et al., 2023 (tumor-cell differentiation) [288]; Zhang et al., 2025 (immune-mediated mechanism) [298]
Tumor microenvironment	Largely tumor-cell-intrinsic; immune-mediated route not prominent in reviewed evidence	Substantial immune-mediated component: PPARγ agonism reprograms TAMs away from pro-tumorigenic M2 phenotype	Zhang et al., 2025 [298]
Pharmacological direction	Both agonism and antagonism produce antitumor effects	Only agonism tested to date; no antagonist data available	Nakao-Ise et al., 2023 (antagonist) [288]; Jang et al., 2022 (agonist) [31]
Genetic background	MYCN amplification determines responsiveness to antagonism (disrupts NMYC/BCL2/BRD4 survival network)	Untested; GBM’s genetic drivers (EGFR, IDH, PGBD5) not yet linked to PPARγ pharmacological direction	Nakao-Ise et al., 2023 [288]
Ligand structure	Structurally distinct agonists diverge sharply: RGZ (modest effect) vs. leflunomide (robust differentiation via PGC-1α)	Not directly compared across ligands within GBM	Krieger-Hinck et al., 2010; Jang et al., 2022 [297]

Together, these four factors suggest that PPARγ’s ‘dual’ function in neuro-oncology is not a genuine contradiction, but the expected output of a receptor whose transcriptional program is conditionally assembled by genetic background, ligand structure, and microenvironmental context rather than fixed to one pharmacological direction.

### Clinical Studies in Neuro-Oncology

In contrast to neurodegenerative disorders, clinical investigations of PPARγ agonists in primary brain tumors remain sparse. The phase I trial NCT01151670 evaluated PIO in patients with primary or metastatic brain tumors undergoing radiotherapy, aiming to assess safety and potential prevention of radiation-induced cognitive dysfunction (RICD) [304]. PIO was administered at 22.5 mg or 45 mg daily beginning one week prior to radiotherapy and continuing for six months post-treatment [304]. Although serious adverse events were rare and tolerability was acceptable, short median survival (5.5 months) and disease progression limited long-term cognitive assessment. Consequently, the protective efficacy against RICD could not be definitively established [304].

The clinical trials NCT00616642, sponsored by Jonsson Comprehensive Cancer Center investigated the efficacy of RGZ in patients with pituitary adenomas, specifically focusing on Cushing’s Disease (ACTH-secreting tumors), although the clinical trial was terminated prematurely due to a lack of therapeutic efficacy and an insufficient number of patients enrolled, compromising statistical power [242]. Despite dose escalation up to 16 mg/day, the treatment achieved neither sustained normalization of urinary free cortisol levels nor significant tumor shrinkage on MRI [305]. This translational gap is supported by recent transcriptomic analyzes revealing significant downregulation of the PPARG gene in tissues from patients with Cushing’s syndrome (including ACTH-producing adenomas and adrenal hyperplasia), suggesting that low receptor density limits the efficacy of agonists such as RGZ in vivo [306].

Taken together, the two available trials fail for distinct reasons that should not be conflated. The RICD trial (NCT01151670) did not demonstrate a negative result so much as an inconclusive one: short median survival (5.5 months) censored long-term cognitive follow-up before efficacy could be assessed, reflecting a trial-design limitation rather than a failure of the mechanism [307]. The Cushing’s disease trial, by contrast, showed a genuine null result despite dose escalation and adequate follow-up. The likely explanation is target-level, not pharmacokinetic: transcriptomic analyses show significant PPARG downregulation in ACTH-secreting adenoma tissue itself [307] meaning the drug may have reached its target with too few receptors available to engage. This distinction matters for future trial design: RICD warrants re-testing with adequate follow-up in patients with better prognosis, whereas Cushing’s disease may require confirming tumor PPARG expression as an inclusion criterion before agonist therapy is attempted again.

## Synthesis and Limitations of Current Evidence

The distinction between a trial-design limitation and a genuine target-level failure, is not unique to neuro-oncology. Table 3 consolidates the clinical trials discussed throughout this review, and considered together, they reveal a similar pattern of clinical underperformance despite consistent preclinical rationale, spanning neurodegenerative, psychiatric, and neuro-oncological conditions. This section examines the factors most likely driving that pattern across disease contexts.

**Table 3:** Advances in clinical trials of PPARγ agonists in diseases affecting the CNS over the past few decades

Posted	Identifiers	Diseases	Drug	Phase	Sponsor
2009	NCT00982202 [197]	AD	PIO	II	National Institute on Aging
2015	NCT01280123 [216]	PD	PIO	II	University of Rochester
2017	NCT00348140 [308]	AD	RGZ	III	GlaxoSmithKline
2017	NCT00835120 [273]	Bipolar Depression	PIO	IV	University Hospitals Cleveland Medical Center
2018	NCT01151670 [305]	Brain Tumors	PIO	I	Wake Forest University Health Sciences
2018	NCT02507219 [309]	Major Depression Disorders	Ibuprofen	II/III	Laureate Institute for Brain Research, Inc.
2019	NCT01931566 [198]	AD	PIO	III	Takeda Pharmaceutical Company
2020	NCT00616642 [242]	Pituitary Tumors	RGZ	II	Jonsson Comprehensive Cancer Center
2020	NCT00612066 [242]	Pituitary Tumors	RGZ	II	Jonsson Comprehensive Cancer Center
2023	NCT04528706 [230]	Adrenoleukodystrophy	Leriglitazone	II	Minoryx Therapeutics, S.L.
2025	NCT03231878 [229]	Adrenoleukodystrophy	Leriglitazone	II/III	Minoryx Therapeutics, S.L.

Across the disease contexts discussed above, preclinical evidence supports a neuroprotective role for PPARγ activation, through improved mitochondrial function, reduced neuroinflammation, and preserved synaptic plasticity [310–312]. However, these findings are not always consistent between studies [312–314]. Effects vary with animal model, dose, and disease stage, and most preclinical models rely on early or preventive PPARγ activation, conditions that do not reflect how patients present clinically, usually after disease onset [216, 313, 314]. This mismatch may explain part of the gap between preclinical promise and clinical outcomes [198, 314].

The role of PPARγ also diverges by cell type, though this has rarely been examined systematically within a single disease context. In neurons, PPARγ appears to exert a tonic, homeostatic function: conditional deletion of neuronal PPARγ enhances stress-induced emotional responses, indicating a baseline regulatory role independent of inflammatory challenge [244]. In microglia, by contrast, PPARγ acts predominantly as an inducible immunomodulator, repressing NF-κB-dependent transcription and shifting cells toward an anti-inflammatory (M2-like) phenotype in response to injury or metabolic stress [31, 315]. In astrocytes, evidence from X-ALD models points to a third, largely bioenergetic role: leriglitazone restores mitochondrial ATP production and reduces oxidative stress in VLCFA-exposed astrocytes, a function more metabolic than immunomodulatory in nature [161].

This divergence, tonic in neurons, inducible-immune in microglia, bioenergetic in astrocytes, likely reflects cell type-specific differences in coactivator and corepressor recruitment at the PPARγ AF-2 domain, which determine whether PPARγ activation engages metabolic, inflammatory, or neurotrophic transcriptional programs in a given cellular context [224, 316]. Disease stage adds a further layer of heterogeneity: the same neuroprotective mechanisms that dominate in early, prevention-oriented paradigms across AD, PD, and X-ALD models appear insufficient once neuroinflammation and network damage become self-sustaining, suggesting that cell type and disease stage jointly determine which PPARγ-dependent program is therapeutically accessible at a given point in disease progression [160, 198, 216, 229, 317].

Clinical trials with classical PPARγ agonists (PIO and RGZ) have largely failed to replicate preclinical benefits, in PD (NCT01280123), AD (NCT00982202, NCT00348140, NCT01931566), and brain tumors (NCT01151670, NCT00616642). Several factors are likely to contribute to this, classical thiazolidinediones have limited blood-brain barrier penetration, which restricts target engagement in the CNS. Second, these agents act as full agonists, producing broad transcriptional activation rather than the more selective, pathway-specific effects that CNS conditions may require. Third, most trials enrolled patients with established or advanced disease, at a stage when neuroimmune and metabolic dysregulation may already be difficult to reverse. In contrast, leriglitazone, developed specifically for CNS penetration, has shown more consistent target engagement and clinical signal in X-ALD [229, 230], supporting the idea that pharmacokinetic optimization, not just target validity, is central to translational success.

Beyond pharmacokinetics, classical agonists carry adverses events that constrain their clinical use, for example weight gain, fluid retention, cardiovascular risk, and, in the case of TZG, hepatotoxicity severe enough to prompt withdrawal from the market [318–320]. These effects limit achievable dosing and treatment duration, independently of whether the CNS mechanism is sound [224, 318–320]. This distinction matters and suggests negative trial results with PIO or RGZ should not be interpreted as evidence against PPARγ as a therapeutic target, but as evidence against a specific pharmacological approach, full, non-selective agonism with poor CNS selectivity [198, 216, 224].

Finally, comparison across trials is limited by inconsistent outcome measures, variable trial duration, and the near-total absence of CNS-specific target engagement biomarkers (e.g., CSF or imaging markers of PPARγ activity) in neurodegenerative and neuro-oncological trials, in contrast to X-ALD studies, which did incorporate such measures [198, 216, 229]. This methodological gap makes it difficult to distinguish trials that failed because PPARγ modulation is ineffective in each condition from trials that failed because the drug never adequately reached or engaged its target in the CNS [198, 216, 224, 229].

## Future Perspectives: Refining the Therapeutic Strategy

Given these limitations in pharmacokinetics, ligand selectivity, and trial design, progress will depend on more precise and context-adapted strategies, rather than on further testing of first-generation agonists. These include the development of CNS-penetrant SPPARMs capable of inducing targeted transcriptional programs, as well as combinatorial approaches engaging multiple PPAR isoforms (e.g., PPARδ/γ). Equally important is the implementation of biomarker-driven patient stratification to identify individuals with inflammatory or metabolic profiles most likely to benefit from intervention.

Advances in translational design should also incorporate multimodal approaches to confirm target engagement, including neuroimaging, peripheral cytokine profiling, and metabolic assessments. In parallel, clinical trials must better align with preclinical evidence by prioritizing early-stage or preventive interventions, where modulation of neuroimmune pathways is more likely to yield meaningful outcomes. Combination therapies targeting complementary mechanisms—such as mitochondrial dysfunction, protein aggregation, or immune signaling—may further enhance therapeutic efficacy.

Within this framework, PPARγ should be conceptualized not as a symptomatic target, but as a central regulator of neuroimmune–metabolic homeostasis. Its therapeutic value likely lies in the long-term modulation of inflammatory tone, metabolic resilience, and cellular plasticity, rather than in acute symptom control. This strategy parallels efforts targeting other pleiotropic receptor systems, such as sigma-1 receptor modulation, which has shown comparable neuroprotective effects through anti-inflammatory and antioxidant mechanisms in preclinical epilepsy models [236], reinforcing the broader rationale for receptor-based metabolic-immune modulation in CNS disorders.

Ultimately, the key challenge is not establishing the biological relevance of PPARγ, but determining how to leverage its regulatory capacity in a manner that is context-specific, stage-dependent, and mechanistically informed. The development of next-generation modulators, coupled with precision-based clinical strategies, will be essential to define whether PPARγ-targeted therapies can achieve meaningful impact across neurodegenerative, psychiatric, and neuro-oncological disorders.

## Data Availability

No datasets were generated or analysed during the current study.
